# Directed assembly of defined oligomeric photosynthetic reaction centres through adaptation with programmable extra-membrane coiled-coil interfaces

**DOI:** 10.1016/j.bbabio.2016.09.002

**Published:** 2016-12

**Authors:** David J.K. Swainsbury, Robert L. Harniman, Natalie D. Di Bartolo, Juntai Liu, William F.M. Harper, Alexander S. Corrie, Michael R. Jones

**Affiliations:** a School of Biochemistry, Medical Sciences Building, University of Bristol, University Walk, Bristol BS8 1TD, United Kingdom; b School of Chemistry, University of Bristol, Cantock's Close, Bristol BS8 1TS, United Kingdom

**Keywords:** Reaction centre, Photosystem, Directed self-assembly, Membrane protein, Coiled-coil, Oligomerisation

## Abstract

A challenge associated with the utilisation of bioenergetic proteins in new, synthetic energy transducing systems is achieving efficient and predictable self-assembly of individual components, both natural and man-made, into a functioning macromolecular system. Despite progress with water-soluble proteins, the challenge of programming self-assembly of integral membrane proteins into non-native macromolecular architectures remains largely unexplored. In this work it is shown that the assembly of dimers, trimers or tetramers of the naturally monomeric purple bacterial reaction centre can be directed by augmentation with an α-helical peptide that self-associates into extra-membrane coiled-coil bundle. Despite this induced oligomerisation the assembled reaction centres displayed normal spectroscopic properties, implying preserved structural and functional integrity. Mixing of two reaction centres modified with mutually complementary α-helical peptides enabled the assembly of heterodimers *in vitro*, pointing to a generic strategy for assembling hetero-oligomeric complexes from diverse modified or synthetic components_._ Addition of two coiled-coil peptides per reaction centre monomer was also tolerated despite the challenge presented to the pigment-protein assembly machinery of introducing multiple self-associating sequences. These findings point to a generalised approach where oligomers or longer range assemblies of multiple light harvesting and/or redox proteins can be constructed in a manner that can be genetically-encoded, enabling the construction of new, designed bioenergetic systems *in vivo* or *in vitro*.

## Highlights

• Reaction centre monomers are engineered to assemble as oligomers *in vivo*.
• A fused coiled coil bundle programs dimer, trimer and tetramer formation.
• Assembled oligomeric reaction centres are structurally and functionally intact.
• Coiled coils can be used to assemble reaction centre hetero-oligomers *in vitro*.
• Addition of two coiled-coil peptides per reaction centre monomer is tolerated.

## Graphical abstract

**Figure:**
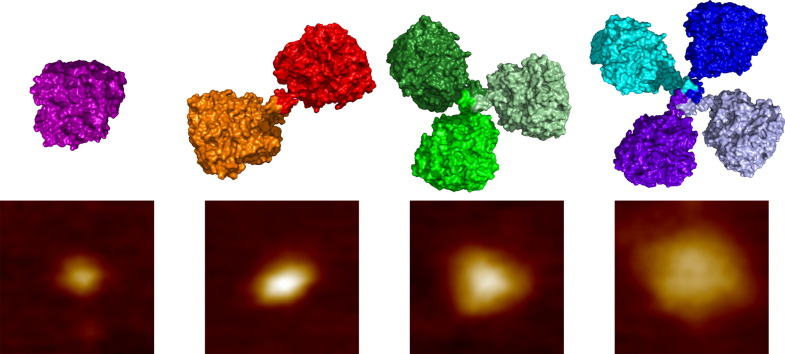

## 1 Introduction

**Fig. 1:**
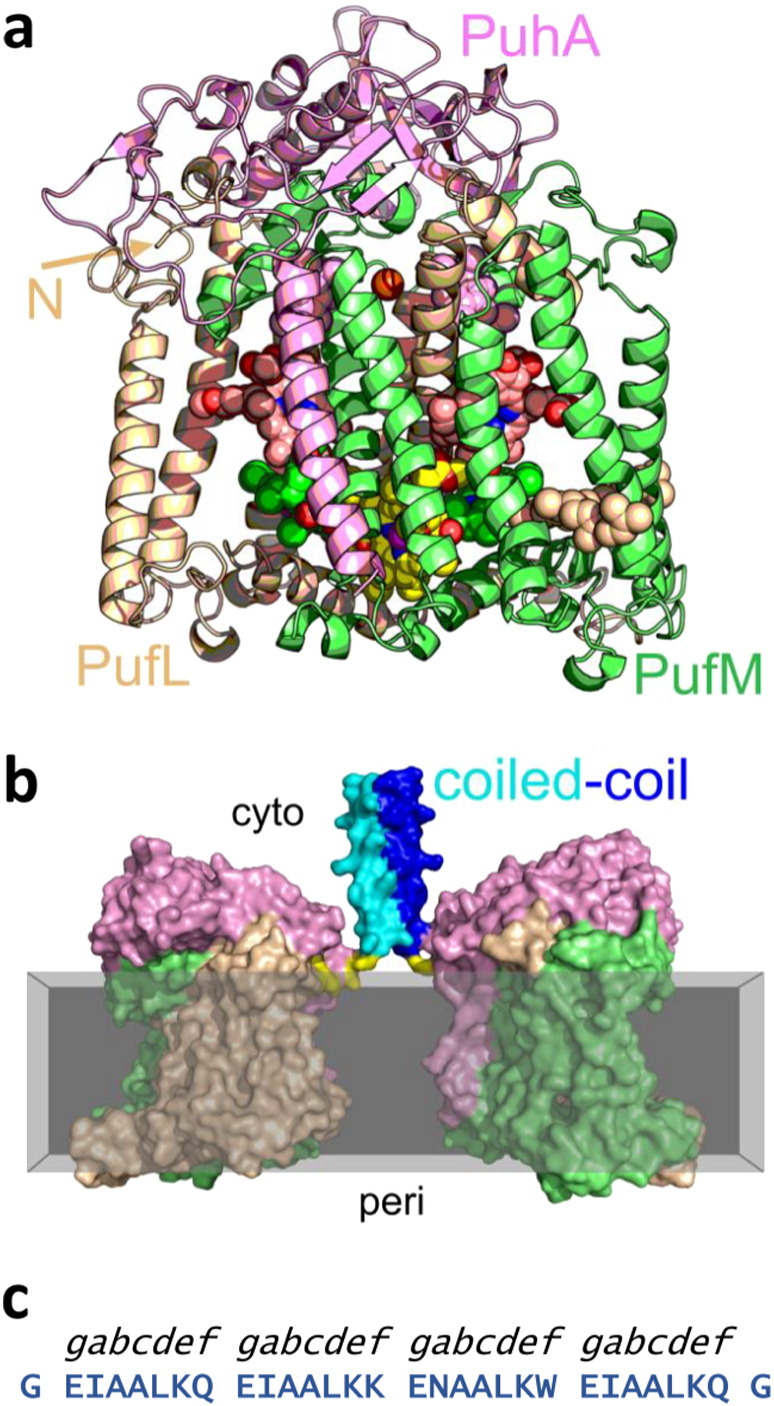
Structure and design. (a) X-ray crystal structure of the *Rba. sphaeroides* reaction centre. The three component polypeptides are shown as beige (PufL), green (PufM) and pink (PuhA) ribbons. PufL and PufM form a largely intramembrane pseudosymmetrical heterodimer that encases ten cofactors, shown as spheres; the cofactors can be seen in detail in Fig. S1 (Supporting information). The arrow indicates the N-terminus of PufL, at the back of the protein and at the surface of the membrane. (b) Molecular model of a designed reaction centre homodimer formed between two coiled-coil α-helices (blue and cyan) each fused to the N-terminus of a PufL by a linker sequence at the membrane interface (yellow). The grey slab represents the approximate position of the membrane, and the cytoplasmic and periplasmic compartments on either side of the membrane are marked. (c) Sequence of the heptad repeat region of the 28 amino acid CC-Di peptide that forms a homodimeric coiled-coil [34], flanked by two glycines. In the broader family of peptides the oligomeric state formed is largely dependent on the identity of the hydrophobic residues at the *a* and *d* positions (and see Fig. S2b and S2e, Supporting information).

One of the underlying principles of the emerging field of synthetic biology is the use of biomolecules as predictable components in engineered, synthetic molecular systems. Bioenergetic proteins that transduce energy and power catalysis are obvious targets for such exploitation. The development of novel systems for solar energy transduction is of particular interest given concerns over fossil fuel use and future energy supply, and there is burgeoning interest in the use of natural reaction centre pigment-proteins in new solar energy technologies [1], [2], [3], [4]. The subject of this report, the reaction centre from the purple bacterium *Rhodobacter* (*Rba.*) *sphaeroides* (Fig. 1a, and see Fig. S1 in Supporting information) facilitates a highly-efficient photochemical charge separation that has been exploited for the construction of prototype solar cells [5], [6], [7], photoelectrochemical cells [8], [9], [10], [11], [12], [13], [14], [15], [16], [17], [18] and biosensors [19], [20]. Reaction centres from oxygenic phototrophs are of particular interest with regard to solar fuel synthesis through water splitting [21], [22] and the powering of catalysis by other redox proteins in non-native, hybrid systems [23], [24]. Alongside the direct exploitation of natural bioenergetic proteins there is growing interest in the *de novo* design of artificial protein-cofactor systems as single proteins or networks [25], and in the interfacing of natural and artificial proteins with man-made materials for energy harvesting, electron transfer and catalysis [3].

The construction of new hybrid bioenergetic systems using combinations of natural components, designed components and synthetic materials raises the challenge of being able to mimic a key aspect of nature, the exertion of a high degree of control over the self-assembly of diverse nanoscale components into a functional system with a defined composition and architecture. This is achieved through information that is genetically encoded and is based on highly specific molecular interactions, including protein-protein interactions that are intrinsically selective, frequently have to be reversible, and are often poorly understood. The effective construction of designed protein assemblies that will carry out new functions with specificity and efficiency similarly requires control to be exerted over how the component parts interact with one another, and an attractive way to achieve this is through genetic encoding. Various strategies have been developed for genetically-directing self-assembly of water-soluble proteins into programmable two- or three-dimensional macromolecular architectures [26], [27], [28], [29], [30], [31]. However, directed self-assembly of integral membrane proteins to form novel and controllable macromolecular architectures is largely unexplored and is obviously of great relevance when considering exploitation of bioenergetic systems that are frequently membrane-centric. The challenges relate to familiar difficulties associated with the expression and handling of integral membrane proteins, a shortage of information on their structures and mechanisms of assembly, and a lack of knowledge at the atomic level of how membrane proteins interact with one another in the complex lipid bilayer.

The work described below stems from observations during recent studies of solar energy conversion by *Rba. sphaeroides* reaction centres incorporated into photoelectrochemical cells [5], [6], [7], [10], [18], [19]. Deposition of this protein on electrode surfaces in such systems is usually achieved by drop-casting or binding from solution, processes which give limited control over protein orientation at the electrode surface and even less control over packing of protein on the electrode surface. Such assembly processes are also inefficient and can be wasteful of (often expensive) biomaterials. This lack of control is in marked contrast to the exquisite precision and efficiency with which the natural photosynthetic membrane is assembled. Given this, we have been exploring genetic strategies for the programmed self-assembly of naturally monomeric reaction centres into fully functional multimers and larger scale assemblies, both *in vivo* and *in vitro*. This has been done with a view to gaining control over the density of packing of protein-embedded electron transfer chains on a conducting surface, adapting the protein for self-assembly onto nanostructured electrode materials, and developing tools for the construction of multicomponent hybrid systems for light powered catalysis. In work leading up to the present study a number of attempts were made to take a first step by inducing the naturally monomeric *Rba. sphaeroides* reaction centre to assemble in a dimeric form through modification of lipid-exposed surfaces of its membrane-spanning helices to introduce the well-known GxxxG helix dimerisation motif [32], [33]. These attempts were not successful despite being informed by modelling based on the X-ray crystal structure of the reaction centre, and highlighted a lack of information on the freedom with which large and complex proteins of this type can mutually interact within the heterogeneous membrane environment.

In the light of this experience, we have explored an alternative strategy in which the cytoplasmically-exposed N-terminus of one of the integral membrane polypeptides of the reaction centre was augmented with an extra-membrane α-helical protein sequence that is known, as a synthetic peptide, to self-associate into a water-soluble oligomeric coiled-coil structure. A schematic is shown in Fig. 1b for the envisaged formation of a reaction centre homo-dimer through modification with an α-helix that will self-associate into a dimeric coiled-coil bundle [34]. The potential advantages of this strategy are that a detailed understanding of the atomic structure of the target integral membrane protein it not required, and it avoids a need to change the structure of the target protein or to attempt to engineer protein:protein interactions within the challenging membrane environment. The use of this as a generic approach also opens up the possibility of engineering hybrid bioenergetic systems in which diverse membrane-embedded or soluble components can be interfaced without having to engineer bespoke protein:protein interfaces for each new combination. On the other hand the introduction of an extra-membrane α-helix with a propensity to self-associate presents a challenge to the molecular process responsible for reaction centre assembly. The *Rba. sphaeroides* reaction centre is formed from three component polypeptides and ten cofactors [35], [36] and, as with a great many bioenergetic protein-cofactor complexes, the processes through which it is assembled are poorly understood.

In contrast, a great deal is known about the assembly of coiled-coils and how such structures can be manipulated through protein engineering. Coiled-coils comprise two or more amphipathic α-helices that associate in a left-handed supercoil [37], [38], [39], [40], [41]. Each α-helix is typically formed from multiple repeats of five polar (P) and two hydrophobic (H) residues in the sequence HPPHPPP, the latter creating a hydrophobic stripe that spirals around each helix. The need to bury this stripe drives the interaction of two or more such α-helices to form a homo- or hetero-oligomeric parallel or antiparallel supercoil. The order of this structure depends on the identity of the polar and hydrophobic amino acids in the heptad repeat, and a great deal of effort has been put into understanding the relationships between sequence and oligomeric structure [42], [43], [44], [45], [46], [47], [48]. Natural coiled-coil proteins are ubiquitous and have many functions. Designed coiled-coil peptides have been used to form diverse structures and biomaterials including oligomers [49], fibres [50], polymers [51] and cages [52]. Libraries of specific oligomerisation partners have been developed [34], [53], [54], [55], [56], [57] and individual coiled-coils have been used for a variety of applications [41], [58] including oligomerisation of water-soluble proteins [59], [60], [61]. Instances exist in nature of extra-membrane coiled-coil domains determining membrane protein oligomerisation, an example being the dimeric Hv1/VSOP voltage-gated H^+^ channel that can be mutated to trimeric and tetrameric forms [62], [63].

In this work we have investigated the use of a family of water-soluble coiled-coil peptides [34] as extra-membrane determinants of reaction centre oligomerisation. As synthetic peptides these form high affinity homo-dimeric, homo-trimeric, homo-tetrameric and hetero-dimeric coiled-coil structures, depending on sequence; the example shown in Fig. 1c forms a homodimer in solution [34]. The aim was to determine whether incorporation of these sequences as tethers would be tolerated by the reaction centre assembly machinery and, if so, would drive artificial homo- or hetero-oligomerisation of reaction centres *in vivo* or *in vitro*. We find that such modification is tolerated without any obvious impact on the structure or functional properties of the reaction centre, and the practicalities of this approach to building new multicomponent bioenergetic systems are explored.

## 2 Materials and methods

### 2.1 Protein engineering, reaction centre purification and spectroscopic analysis

DNA sequences encoding a coiled-coil α-helix and linker were inserted immediately after the start codon of *pufL* or *pufM* (see additional Methods and Fig. S2 in Supporting information for details). This was done in plasmid pUCLMt, which is a derivative of pUC19 containing a *Xba*I-*Bam*HI restriction fragment encompassing a native copy of *pufL* and a copy of *pufM* modified to introduce a ten residue poly-histidine tag at the C-terminus of PufM for purification (see additional Methods in Supporting information). The resulting *Xba*I-*Bam*HI fragments were shuttled into plasmid pRKEH10D [64], which is a derivative of broad-host-range vector pRK415 containing a 6.2 kb *Eco*RI-*Hin*dIII fragment encoding *pufQLM*. The resulting plasmids were transferred to *Rba. sphaeroides* strain DD13 as described previously [64], [65]. Transconjugant strains contained reaction centres as the sole pigment-protein complex in the bacterial cell, enabling assessment of reaction centre expression levels through absorbance spectra of cell cultures or preparations of photosynthetic membranes. Procedures used for the isolation of photosynthetic membranes and the purification of reaction centres are given in Supporting information.

Measurements of the formation and recombination of P^+^ Q^−^ were performed as described in detail previously [66]. Oxidation of cyt *c* by reaction centres was monitored at 550 nm using a Cary60 spectrophotometer connected to an external CUV 1 cm cuvette holder (Ocean Optics) *via* a pair of optical fibres. Photo-oxidation was induced by illumination with a HL-2000-FHSA shutter controlled white light source (Ocean Optics), delivering *via* an optical fibre a light intensity of approximately 2.5 mW cm^− 2^ at the cuvette surface at 90° to the pulsed measuring beam. Samples comprised 0.5 μM reaction centres in 20 mM Tris (pH 8)/0.1% LDAO supplemented with 50 μM reduced cyt *c* and 50 μM UQ_0_ in a 3 × 3 mm fluorescence cuvette (Hellma). Four transients were recorded for each sample and averaged.

### 2.2 Analytical gel filtration

A Superdex 200 10/300 GL column (GE Healthcare) was pre-equilibrated with 20 mM Tris (pH 8)/200 mM NaCl/0.04% (w/v) n-dodecyl β-D-maltopyranoside (DDM – denoted RC-DDM buffer) at 4 °C. A 100 μL aliquot of 10 μM wild-type (WT) or homo-oligomeric reaction centre, 10 μM individual L-htDiA or L-htDiB reaction centre, or a mixture of 5 or 25 μM of each of the latter reaction centres was injected onto the column and eluted over 1.5 column volumes whilst monitoring absorbance at 365 nm. All samples were incubated for approximately 1 h at room temperature before injecting onto the column. A calibration curve was generated by running standards over a 12–200 kDa range (Sigma Aldrich) in the same buffer.

### 2.3 Analytical ultracentrifugation

Reaction centres were exchanged into 20 mM Tris (pH 8) containing 200 mM NaCl and 0.35% (w/v) pentaethylene glycol monooctyl ether (C_8_E_5_) by three rounds of ten-fold dilution followed by re-concentration using 100,000 MWCO Vivaspin 500 centrifugal concentrators (Vivaproducts, Inc.). Values for buffer density and viscosity were calculated in Sednterp [67] ignoring the contribution by C_8_E_5_ as its density matches that of water [68]. Mass and values of partial specific volume (vbar) for the proteins were calculated in Sednterp and modified to include published parameters for the reaction centre cofactors [69].

For sedimentation velocity experiments 2.5 μM reaction centres were loaded into two channel sector-shaped cells and centrifuged at 30,000 rpm at 25 °C for 5 h in an An50-Ti rotor in a Beckman XL-I analytical ultracentrifuge. Absorbance profiles were collected every 2 min at 365 nm with a radial step size of 0.003 cm. Data were analysed as a continuous sedimentation coefficient distribution c(s) in Sedfit from which molecular weights were estimated [70].

For equilibrium experiments three samples of 1 μM of each reaction centre were loaded into channel equilibrium cells and run at 25 °C for 20 h at 5000, 8000, 11,000 or 14,000 rpm in an An50-Ti rotor in a Beckman XL-A analytical ultracentrifuge. Five scans were collected with a step size of 0.003 cm at 365 nm for each sample. All data were analysed using Ultrascan II [71] using data from all three cells at speeds 8000, 11,000 and 14,000 rpm for WT reaction centres and 5000, 8000 and 11,000 rpm for the oligomeric reaction centres. Curves were fitted to a two-component ideal model and subjected to 10,000 rounds of Monte-Carlo analysis with 8% added noise.

### 2.4 Sucrose gradient ultracentrifugation

Sucrose step gradients were prepared by sequentially layering 2 mL volumes of solutions of 25%, 23.75%, 22.5%, 21.25% and 20% sucrose (w/v) in RC-DDM buffer [72]. Prepared gradients were loaded with 400 μL of 13.9 μM WT or homo-oligomeric reaction centres, or with a mixture of 6.95 μM of each of the L-htDiA and L-htDiB reaction centres incubated for approximately 1 h at room temperature before loading. Gradients were centrifuged at 247,000 RCF for 20 h at 4 °C in a Sorvall TH641 rotor.

### 2.5 Dynamic light scattering

Measurements were carried out on solutions of 2 μM reaction centre in 20 mM Tris (pH 8)/200 mM NaCl in a 200 μL micro cuvette (Hellma) in a Zitasizer Nano ZS (Malvern Instruments). Correlation curves were produced from three sets of twelve spectra and processed using the Zitasizer software package.

### 2.6 Atomic force microscopy

Solutions of 3.125 nM WT or oligomeric reaction centres were prepared in 5 mM Tris (pH 8)/0.02% DDM. A 10 μL aliquot was dried onto a 1 × 1 cm square of freshly cleaved muscovite mica for approximately 30 min. Each surface was investigated using a MultiMode 8 atomic force microscope (Bruker, CA, USA) under ambient conditions. Deformation of the sample was minimised using non-resonant PeakForce control to maintain the minimal level of force interaction required to resolve the surface in the range < 100 pN. A single cantilever was used across all four samples in a single day to remove the possibility of tip-shape being responsible for the differences in small scale conformation. The cantilever used (ScanAsyst-Air-HR, Bruker, CA, USA) had a nominal 2 nm tip radius and 0.4 N/m spring constant.

### 2.7 Reconstitution of reaction centres into liposomes

Reaction centres were reconstituted into 400 nm diameter 1,2-dioleoyl-*sn*-glycero-3-phosphocholine (DOPC) liposomes. DOPC (100 mg) was solubilised in 400 μL 1:1 (v:v) CHCl_3_:MeOH and the solution freeze dried overnight. For some reconstitutions rhodamine-1,2-dioleoyl-*sn*-glycero-3-phosphoethanolamine (rhodamine-DOPE) was included at a final concentration of 0.05% to track the liposome fraction during flotation assays. Freeze dried lipids were resuspend at 100 mg mL^− 1^ in 20 mM Tris (pH 8.0)/200 mM NaCl and incubated at room temperature for 1 h with continuous stirring. Liposomes with a diameter of 400 nm were then formed by extrusion through a Nuclepore Track-Etch polycarbonate membrane (Whatman).

Reaction centres purified by nickel affinity chromatography using LDAO as the solubilising detergent (see Supporting information) were detergent exchanged into octyl β-d-glucopyranoside (OG) by gel filtration in 20 mM Tris (pH 8)/200 mM NaCl/0.88% OG (RC-OG buffer) on a Superdex 200 16/600 column (GE Healthcare), and used for subsequent experiments as concentrated solutions in RC-OG buffer.

WT reaction centres were combined with liposomes at a molar ratio of 5000:1 lipid:protein and an absorbance spectrum taken to check the starting reaction centre concentration. To promote reconstitution, OG was removed by dialysis or using BioBeads. For the former, 500 μL aliquots of reaction centre/liposome mixture were placed in Maxi GeBAflex-tubes with a 12–14 kDa MWCO (Generon) and dialysed at 4 °C against 600 mL 20 mM Tris (pH 8)/200 mM NaCl. The buffer was changed twice at hourly intervals, with a final incubation overnight. For the latter, 100 mg BioBeads SM-2 resin (Bio-Rad) pre-soaked in 20 mM Tris (pH 8)/200 mM NaCl were incubated with the reaction centre/liposome mixture at room temperature for 1 h, the BioBeads removed by centrifugation, and the procedure repeated twice. After either procedure an absorbance spectrum was recorded to quantify the reaction centre concentration to account for any losses, which were typically 0–10% and never > 20%. There did not appear to be any significant difference in the efficiency of reconstitution achieved using these two procedures.

Reconstitution efficiency was quantified by a sucrose flotation assay. For the majority of assays, 200 μL of OG-depleted reaction centre/proteoliposome mixture was combined with 200 μL 60% (w/v) sucrose in 20 mM Tris (pH 8)/200 mM NaCl and pipetted into the bottom of a 2.2 mL thin wall polyallomer ultracentrifuge tube. This was then overlaid with 1.6 mL of 15% sucrose (w/v) in 20 mM Tris (pH 8)/200 mM NaCl, and this in turn was overlaid with 200 μL 20 mM Tris (pH 8)/200 mM NaCl. In both cases care was taken to avoid mixing. Tubes were centrifuged at 200,000 ×* g* for 1 h at 25 °C in a TLS55 swing out rotor in a Beckman bench top ultracentrifuge. Each sucrose step gradient was carefully deconstructed from the top into six equal fractions and absorbance spectra recorded. The last fraction removed from each tube was agitated vigorously to resuspend any unreconstituted reaction centres pelleted at the bottom of the tube. For some flotation assays (examples in Fig. 6a) the volume of each component was scaled-up approximately five-fold in ~ 12 mL clear polycarbonate ultracentrifuge tubes and gradients were centrifuged in a Sorvall TH-641 swing-out bucket rotor at 250,000*g* for 1 h at 25 °C.

Co-migration of the bulk of the reaction centre population with the liposome fraction in sucrose flotation assays was quantified by absorbance spectroscopy using the characteristic reaction centre absorbance bands between 650 and 950 nm and the 570 nm band of rhodamine-DOPE. Raw spectra (*e.g.* see Fig. 6b) were corrected for background scatter before estimating absorbance from reaction centres or rhodamine-DOPE. The efficiency of reconstitution was determined by comparing the concentration of reaction centres in the liposome-containing fractions of the deconstructed sucrose step gradients with that in the starting material prior to dialysis.

### 2.8 Molecular dynamics simulations

These were carried out as described in detail in Supporting information.

## 3 Results and discussion

### 3.1 Engineering and assembly of membrane protein/coiled-coil fusions *in vivo*

**Fig. 2:**
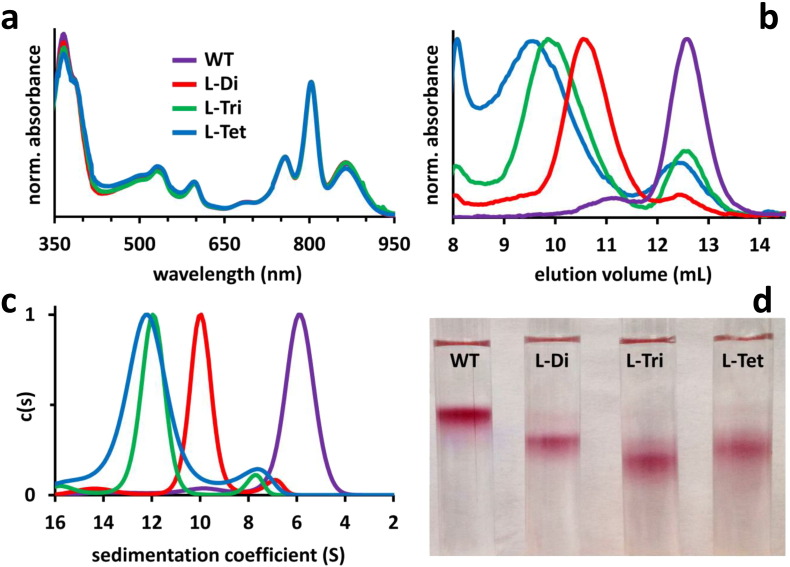
Determination of the oligomeric state of PufL coiled-coil modified reaction centres. (a) Absorbance spectra of purified WT or PufL-modified reaction centres, normalised to the same absorbance at 800 nm. (b) Analytical gel filtration of multimer fractions from preparative gel filtration. (c) Sedimentation coefficients determined by sedimentation velocity AUC. (d) Sucrose density gradient ultracentrifugation. The red colouration of the protein band arises principally from the single reaction centre carotenoid. For panels b and c colour coding is as for panel a.

The *Rba. sphaeroides* reaction centre comprises three polypeptides two of which, PufL and PufM, form a pseudosymmetrical membrane-embedded structure that provides a scaffold for the electron transfer cofactors (Fig. 1a and Fig. S1 in Supporting information). To attempt to induce oligomerisation, the N-terminus of the reaction centre PufL polypeptide (arrow labelled N in Fig. 1a) was modified to encode a six amino acid water-soluble linker preceded by a 31 amino acid α-helical sequence that is known to self-associate into either a dimeric, trimeric or tetrameric coiled-coil [34] (see Methods and Fig. S2a,b in Supporting information). The design of these constructs was informed by the X-ray crystal structures of the reaction centre [73] and the three coiled-coil modules [34], and molecular dynamics simulations of idealised structures that the resulting fusion proteins could form in a bilayer membrane (see below). The modified reaction centre genes were expressed in a strain of *Rba. sphaeroides* that lacked light-harvesting complexes [65], and the impact of modification on reaction centre assembly could therefore be assessed through the distinctive absorbance spectra of the bacteriochlorin cofactors of the reaction centre at 756 nm, 805 nm and 865 nm (Fig. 2a). An initial screen of the spectra of intact photosynthetic membranes (data not shown) showed that addition of the dimer and trimer coiled-coil sequences had not affected reaction centre expression levels relative to an unmodified control, and these PufL-modified reaction centres were denoted L-Di and L-Tri, respectively. However, addition of the tetrameric coiled-coil reduced assembly of intact reaction centres below detectable levels, absorbance spectra comprising a single band at 760 nm characteristic of unincorporated bacteriochlorin pigment resulting from protein misfolding (data not shown). Accordingly, the gene modification was revised to extend the linker between the tetrameric coiled-coil sequence and the reaction centre body from six to ten residues, and then to 19 residues. The second of these changes resulted in a markedly improved level of reaction centre expression (denoted L-Tet), although there was still some evidence of free bacteriochlorin pigment in the spectra of intact membranes (data not shown).

### 3.2 Engineered coil-coils induce oligomerisation of functionally-intact reaction centres

Wild-type (WT) and PufL-modified reaction centres were isolated from photosynthetic membranes and purified by nickel affinity chromatography followed by preparative gel filtration (see Methods). WT reaction centres ran as a single band during gel filtration but each of the three modified reaction centres ran as two partially overlapping bands with a yield of > 80% of a higher molecular weight species for L-Di and L-Tri and ~ 30% for L-Tet. Fractions corresponding to this heavier population were pooled and the reaction centres concentrated for all subsequent analysis; the cofactor absorbance spectrum of each of these complexes was indistinguishable from that of the WT reaction centre implying correct assembly and folding of individual reaction centre pigment-proteins (Fig. 2a).

Analytical gel filtration of the resulting concentrated reaction centres revealed a reduction in retention volume corresponding to an increase in mass in the order WT < L-Di < L-Tri < L-Tet (Fig. 2b); estimated masses are listed in Table 1. For each modified reaction centre the dominant species was the oligomer but there was some contamination from a fraction corresponding to monomeric reaction centres. Analytical ultracentrifugation (AUC) was also used to obtain information on mass, velocity measurements producing an increase in sedimentation coefficient that again was in the order WT < L-Di < L-Tri < L-Tet (Fig. 2c); estimated masses are shown in Table 1. Equilibrium AUC measurements (Fig. S3, Supporting information), which were expected to produce the most accurate estimates, revealed a 1.8-fold increase in mass for L-Di, a 2.9-fold increase for L-Tri and a 4.0-fold increase for L-Tet (Table 1).

Reaction centre oligomers from gel filtration were analysed by sucrose gradient ultracentrifugation (Fig. 2d). Pigmented L-Di and L-Tri reaction centres showed clear size increases relative to WT monomers, with L-Tet reaction centres consistently migrating to a position between those of L-Di and L-Tri reaction centres. A similar trend was seen in measurements of dynamic light scattering, with L-Tet reaction centres producing a value for hydrodynamic radius (11.1 ± 6.0 nm) that was intermediate between those for L-Di (9.0 ± 4.3 nm) and L-Tri reaction centres (12.7 ± 5.7 nm), with all three being larger than WT monomers (7.5 ± 3.0 nm). The anomalous behaviour of L-Tet reaction centres in these measurements is likely to reflect the fact that the data from these techniques is influenced by molecular shape and density as well as mass.

### 3.3 Effect of oligomerisation on reaction centre function

**Fig. 3:**
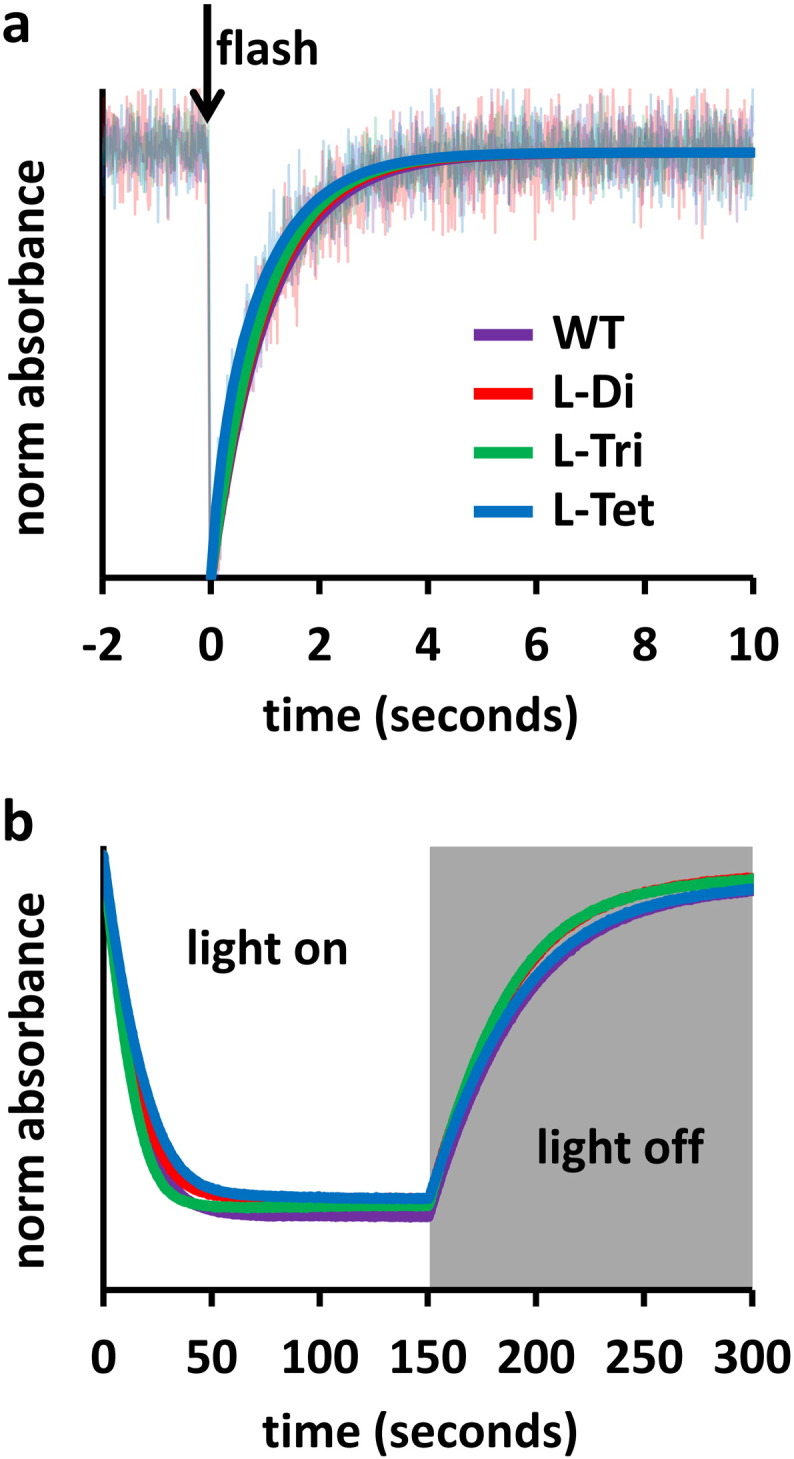
Functional integrity of PufL-modified reaction centres. (a) Kinetics of P^+^ Q_B_^−^ charge recombination monitored at 865 nm. Normalised averages from eight individual kinetic traces are shown in faded lines overlaid with fits to a single exponential decay function. Position of the excitation flash is indicated by a downward arrow. (b) Oxidation of cyt *c* by reaction centres during 150 s of continuous illumination monitored at 550 nm. The dark decay is due to re-reduction of cyt *c*. All traces the average of four individual measurements.

To establish whether tethering reaction centres together into defined oligomers had affected their functional properties, two spectroscopic assays were carried out. Reaction centre internal function was assayed by photo-inducing charge separation between the primary electron donor pair of BChls (P) at the periplasmic side of the protein and the acceptor quinones (Q) on the opposite side and then monitoring the rate of recombination of the resulting P^+^ Q^−^ radical pair (grey dashed arrow in Fig. S1). The amplitude and kinetics of the associated absorbance changes reported on the yield of charge separation and the occupancy of the Q_B_ binding site by ubiquinone. As shown in Fig. 3a, oligomerisation had no significant effect on the yield of P^+^ formation or on the kinetics of P^+^ Q^−^ decay.

The ability of photo-oxidised reaction centres to oxidise cyt *c* was measured as described in Materials and methods. Again, on the basis of this assay (Fig. 3b) it was possible to conclude that inducing oligomerisation did not significantly affect the ability of the tethered reaction centres to bind and oxidise cyt *c*.

In line with these findings that interactions with dissociable ubiquinone and cyt *c* were not perturbed by oligomerisation, preliminary experiments employing photoelectrochemical cells of a similar design to that described previously [10], [19] showed that oligomers of the L-Di, L-Tri and L-Tet reaction centres were capable of generating a photocurrent when interfaced with a gold electrode using water-soluble ubiquinone-0 and cyt *c* as mediators (data not shown). This characterisation is ongoing and a detailed account will be published elsewhere.

### 3.4 Single reaction centre oligomers have distinctive molecular shapes

**Fig. 4:**
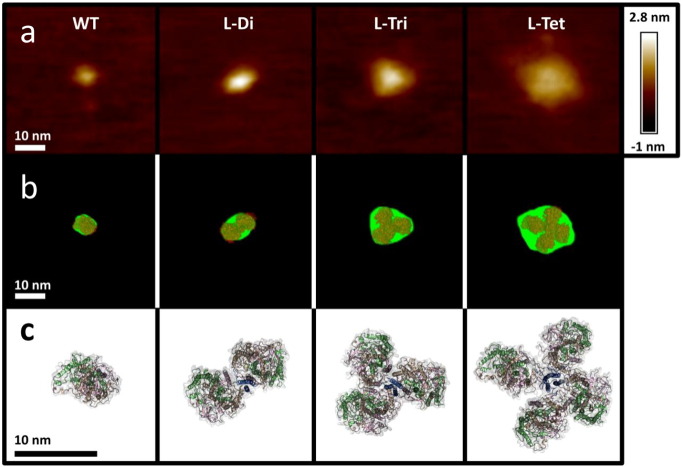
Atomic force microscopy and molecular modelling of oligomeric reaction centres. (a) AFM images of individual WT and PufL-modified reaction centres deposited on mica. (b) Outer contour of the AFM images at a threshold above the background roughness of the mica (green) overlaid with surface-representations of the molecular models shown in c. The model was manually aligned with the contour in each case. (c) Molecular models of WT or multimeric reaction centres generated in GROMACS 5.0 and viewed perpendicular to the membrane from the PuhA (cytoplasmic) side. Molecules are shown with a semi-transparent grey surface and polypeptides in beige (PufL), green (PufM) and pink (PuhA). The fused coiled-coil peptide and linker are shown in blue.

Purified reaction centre oligomers were dried on mica surfaces and examined by atomic force microscopy (AFM). WT reaction centres presented a slightly elliptical profile (dimensions 6.3 × 7.8 nm) (Fig. 4a, left), consistent in size and shape with a monomeric reaction centre viewed perpendicular to the membrane (Fig. 4c, left). L-Di reaction centres were consistently larger and more elliptical (6.5 × 12.3 nm), consistent with two reaction centres located side-by-side, whereas L-Tri and L-Tet reaction centres were found to be larger still and many individual complexes had distinctive three- or four-sided outlines when imaged by AFM (dimensions of the complexes shown in Fig. 4a are 10.3 × 12.3 nm and 11.9 × 12.2 nm, respectively). The images shown in Fig. 4a were representative of the bulk of complexes seen for each reaction centre oligomer; Figs. S4 and S5 in Supporting information shows wide-field images in which multiple examples of each oligomer were observed within a 500 × 500 nm area.

To confirm that the dimensions of the macromolecules imaged by AFM were consistent with assemblies of between one and four reaction centres, molecular outlines calculated by analysis of the AFM images (Fig. 4b, green) were overlaid with the atomic structure of the WT reaction centre or energy-minimised molecular models of tethered homo-dimeric, -trimeric and -tetrameric coiled-coil modified reaction centres embedded in a 1-palmitoyl-2-oleoyl-*sn*-glycero-3-phosphocholine (POPC) bilayer (Fig. 4c). A detailed account of this molecular modelling is given in Supporting information and Figs. S6–S10. As shown in Fig. 4b, the dimensions and gross geometries of the final energy minimised models viewed perpendicular to the plane of the membrane were, in each case, consistent with the molecular envelopes calculated from AFM images.

**Fig. 5:**
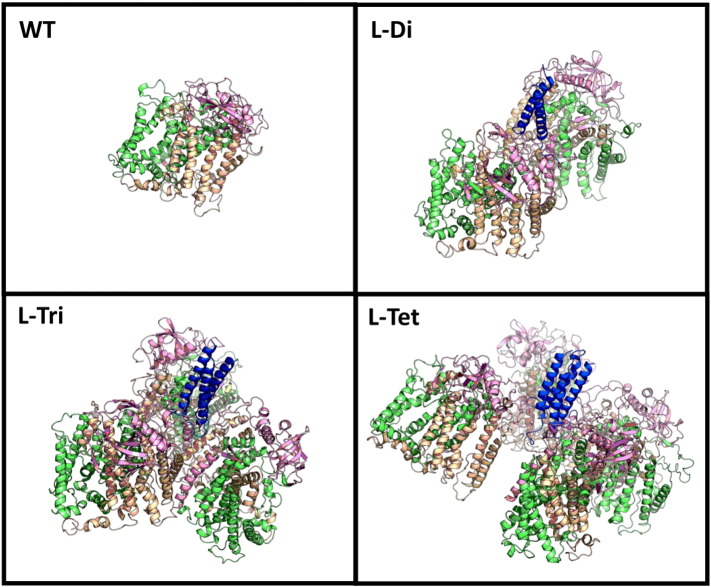
Molecular dynamics simulations of WT or PufL-modified multimeric reaction centres. Final models from one of three replicate simulations for each complex. Individual polypeptides are shown in beige (PufL), green (PufM), pink (PuhA) or blue (coiled-coil peptide and linker fused to PufL). Models of multimers are oriented to provide the best view of the coiled-coil.

As explained in detail in Supporting information, energy minimisation produced compact model structures for each of the reaction centre oligomers (Fig. 5). However no specific molecular interactions between adjacent reaction centres were identified, the designed coiled-coil interface appearing to be the sole determinant of the final oligomeric structure (blue in Fig. 5). The coiled-coil bundle was initially modelled perpendicular to the plane of the membrane, but by the end of each simulation the angle it was sitting at relative to the membrane varied somewhat between independent simulations (Fig. S10, Supporting information). This suggested that there is considerable flexibility in the linker regions, which eliminates the requirement for precise positioning of the coiled-coil relative to the reaction centre, as was intended in the initial design. The coiled-coils showed no propensity for interacting with the lipid bilayer, which was expected due to the soluble nature of these peptides when correctly assembled, again conforming to the desired properties of the fused peptides.

### 3.5 Reaction centre oligomers could be reconstituted into bilayers

**Fig. 6:**
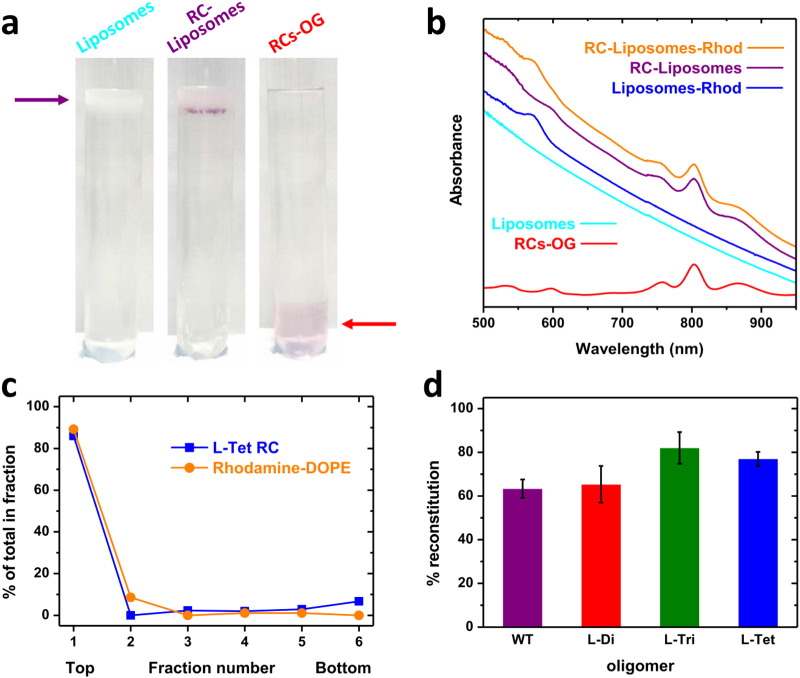
Reconstitution of reaction centres into DOPC liposomes. (a) Sucrose step gradients showing co-migration of reaction centres and liposomes diagnostic of reconstitution. (b) Absorbance spectra of reaction centres, liposomes and reaction centre proteoliposomes. (c) Location of proteoliposomes and L-Tet reaction centres in six fractions isolated from a gradient of the type shown in a. Percentage reconstitution for WT and PufL-modified oligomeric reaction centres. Error bars represent standard error of the mean.

Purified oligomeric reaction centres that had been detergent exchanged into octyl β-D-glucopyranoside (OG) could be reconstituted into an artificial lipid bilayer comprising 1,2-dioleoyl-*sn*-glycero-3-phosphocholine (DOPC) using the same reconstitution protocol as for WT monomeric reaction centres (see Methods). Reconstitution was assessed using a sucrose flotation assay (Fig. 6a, left), purple-coloured reaction centres either floating at the 0/15% interface near the top of a sucrose step gradient if successfully reconstituted into liposomes (Fig. 6a, purple arrow) or migrating to the 30% sucrose fraction at the bottom of the gradient when in detergent (OG) solution (Fig. 6a, red arrow). Consistently, in the presence of reaction centres the entire liposome band acquired a purple colouration with a tight layer of heavily pigmented proteoliposomes at the bottom of the band (Fig. 6a, middle).

The efficiency of reconstitution was assayed spectrophotometrically (see Methods), 0.05% rhodamine-1,2-dioleoyl-*sn*-glycero-3-phosphoethanolamine (rhodamine-DOPE) being used to track and quantify the liposome fraction through its absorbance band at 570 nm (Fig. 6b). In all cases the majority of reaction centres were found in the top two fractions taken from the gradient which also corresponded to the fractions containing liposomes (see sample data for L-Tet reaction centres in Fig. 6c). This distribution was confirmed by dot blots using antibodies against the His_10_ tag on the reaction centre (data not shown). Approximately 65% of the starting protein was reconstituted into the liposome fraction for the WT and L-Di reaction centres, and this value was consistently higher for the L-Tri (~ 80%) and L-Tet reaction centres (~ 75%) (Fig. 6d). This difference was seen in reconstitutions involving both BioBeads and dialysis to remove the OG detergent.

A trypsin digestion assay (not shown) gave some indication that the presence of the coiled-coil module affected the sidedness of the reconstituted oligomeric reaction centres, and this is the subject of ongoing investigations (see Supporting information for a discussion).

### 3.6 Reaction centre hetero-oligomers can be assembled *in vitro*

**Fig. 7:**
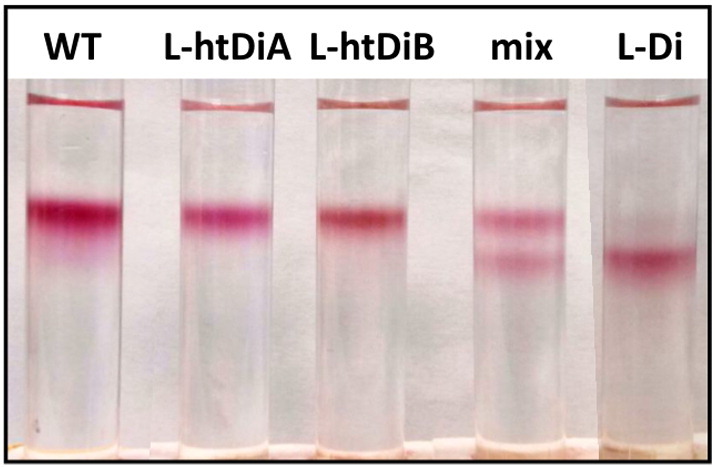
Evidence for *in vitro* heterodimerisation of PufL-modified reaction centres. Migration of a 1:1 mixture of L-htDiA and L-htDiB reaction centres (mix), compared to WT, homodimeric L-Di and individual L-htDiA and L-htDiB reaction centres. The yield of heterodimer was approximately 50% by band intensity.

We also examined whether oligomerisation could be induced *in vitro*, as this would be required, for example, to assemble a hybrid system comprising light harvesting and/or redox proteins from different sources. To achieve this, two complementary α-helical sequences (Fig. S2b, Supporting information) known to assemble as a heterodimeric coiled-coil [57] were engineered at the N-terminus of PufL and expressed in separate strains of *Rba. sphaeroides*. The resulting L-htDiA or L-htDiB reaction centres were purified separately and then mixed as described in Methods. Either individual modified reaction centre was monomeric, migrating on sucrose density gradients as a single band in a manner similar to monomeric WT reaction centres, and with no pigmentation at the position expected for dimeric reaction centres (Fig. 7). However, an equimolar mixture of the two yielded two coloured bands indicative of a roughly 50:50 mixture of monomers and dimers (“mix” in Fig. 7). Despite the clear resolution of heterodimers by this technique, analytical gel filtration failed to resolve dimers, most protein migrating as monomer or a fraction that was intermediate between the expected elution volumes for monomer and dimer. This suggests any dimer formed dissociates upon dilution as it runs ahead of the bulk-protein (data not shown). Taken together, these data suggest that heterodimers formed with a significantly lower affinity than homodimers. Nevertheless, the data provided proof of principle that it is possible to assemble hetero-oligomers *in vitro*, and we are currently exploring how the coiled-coil module can be modified to increase the yield and stability of heterodimers.

### 3.7 Reaction centre oligomerisation can be induced through modification of the symmetrical PufM polypeptide

To test whether oligomerisation could be induced using a different attachment point for the coiled-coil, the effects of modifying the N-terminus of the pseudosymmetrical PufM polypeptide were also explored. The coiled-coil sequences (Fig. S2e, Supporting information) were identical to those used for the PufL series of reaction centres, but a 16 amino acid linker was used as the N-terminus of PufM is somewhat more occluded by surrounding protein than its PufL counterpart (see Supplementary Information for details on the protein engineering).

As with the PufL series, the α-helix attached to PufM induced reaction centres to self-assemble as a homo-dimer, -trimer or -tetramer, depending on its sequence, as evidenced by analytical gel filtration (Fig. S11b, Supporting information) and sucrose density ultracentrifugation (not shown). For the latter the migration of the band corresponding to tetrameric reaction centres at a position intermediate between that of dimers and trimers seen for the PufL modified reaction centres (Fig. 2d), was also seen for the PufM modified reaction centres. The absorbance spectra of the purified PufM-modified reaction centres were essentially identical to that of the WT reaction centre (Fig. S11a, Supporting information). Functional assays showed that these PufM modified reaction centres were also unaffected by tethering together into an artificial oligomer (Fig. S12a,b, Supporting information).

### 3.8 Do programmed membrane protein oligomers assemble *in vivo* or *in vitro*?

The assembly of reaction centre oligomers through the formation of an engineered extra-membrane coiled-coil bundle could occur (1) concurrent with assembly of reaction centre monomers from the three component polypeptides and ten cofactors, (2) following the assembly of individual monomers in the bacterial membrane or (3) following detergent extraction of monomeric reaction centres from the membrane. The finding that the expression level of reaction centres modified with a tetrameric coiled-coil α-helix was dependent on the length of the linker sequence suggests that the first of these was most likely, the implication being that early formation of the coiled-coil and the tethering of four PufL proteins at their N-termini interferes with the assembly of monomers if the linker sequence is insufficiently long. Were the formation of tetramers to occur after that of monomers, or after detergent extraction, then one might expect to see a normal level of reaction centre monomers rather than evidence of impaired reaction centre monomer assembly, given that the α-helical N-terminal extension *per se* seems to be tolerated.

To further investigate how the length of the linker influenced the ability of L-Tet reaction centres to assemble as tetramers, the molecular dynamics simulations described above employing the final 19 residue linker required for tetramer assembly were also carried out with the short, six residue linker that prevented assembly of either tetrameric or monomeric L-Tet reaction centres. It was found that the initial model could be assembled without having to distort the geometries or structures of either the coiled-coil or four reaction centre monomers, and when simulations were run a more compact structure was obtained (data not shown) that was similar to that described for tetramers with the long 19 residue linker (Fig. 5). The conclusion drawn from this is that the required length of the linker is determined not only by the distance between the coiled-coil and the tethered membrane protein monomers in the final structure, but also by the space needed to assemble the membrane protein monomers when individual PufL chains are tethered together at their N-terminus. The process of reaction centre assembly is complex, requiring the bringing together of three polypeptides and ten cofactors, and involves a number of poorly-characterised assembly factors that are integral membrane proteins. Evidently sufficient space for this process is provided by a six residue linker when reaction centres are tethered in a dimeric or trimeric geometry, but assembly of tetramers requires the greater flexibility that a longer linker offers. We note that even with this extended 19 residue linker there was evidence of some impairment of reaction centre assembly in cells and intact membranes of the strain with the L-Tet reaction centre (see above), suggesting that an even longer linker may have been optimal.

### 3.9 Reaction centres tolerate the presence of two pseudosymmetrical coiled-coils

Just as the addition of a single extra-membrane coiled-coil-forming α-helix provides a means of inducing oligomerisation, so the addition of two α-helices at separate sites could in principle enable the formation of longer-range self-assembled arrays of one or more integral membrane proteins. For a protein comprising a single polypeptide chain this could be through modification of both the N- and C-termini, provided they are accessible. In the case of a pseudosymmetrical multi-chain protein such the reaction centre there are multiple potential attachment sites on either side of the membrane (as for all three component polypeptides the N- and C-termini are on opposite sides of the membrane). Depending on the types of homo or hetero coiled-coils employed and their attachment sites, it can be envisaged that an assembled array could have a range of 2-D or 3-D morphologies. In the particular case of the *Rba. sphaeroides* reaction centre, the axis of two-fold symmetry would also affect the morphology of a potential array. As our experience is that coiled-coil bundles do not form with a 100% yield, the large scale assembly of a 2-D array *in vivo* would probably be compromised by the presence of sub-populations of monomeric coiled-coil modified membrane proteins, or those associating at only one of the modified positions. However if component parts of an array were assembled independently of one another, through expression in different bacterial strains or cell lines, then it might prove possible to assemble a long range array *in vitro* by mixing compatible components that interact through a hetero-oligomeric coiled-coil that only forms on mixing. The example illustrated in Fig. S13 (Supporting information) envisages how mixing of two varieties of purified reaction centre homo-trimers (green and yellow) could result in self-assembly of a two-dimensional array if they were also modified at a suitable position with helices that will form a hetero-dimeric coiled-coil interaction (blue and cyan) to link each trimer to three complementary trimers.

**Fig. 8:**
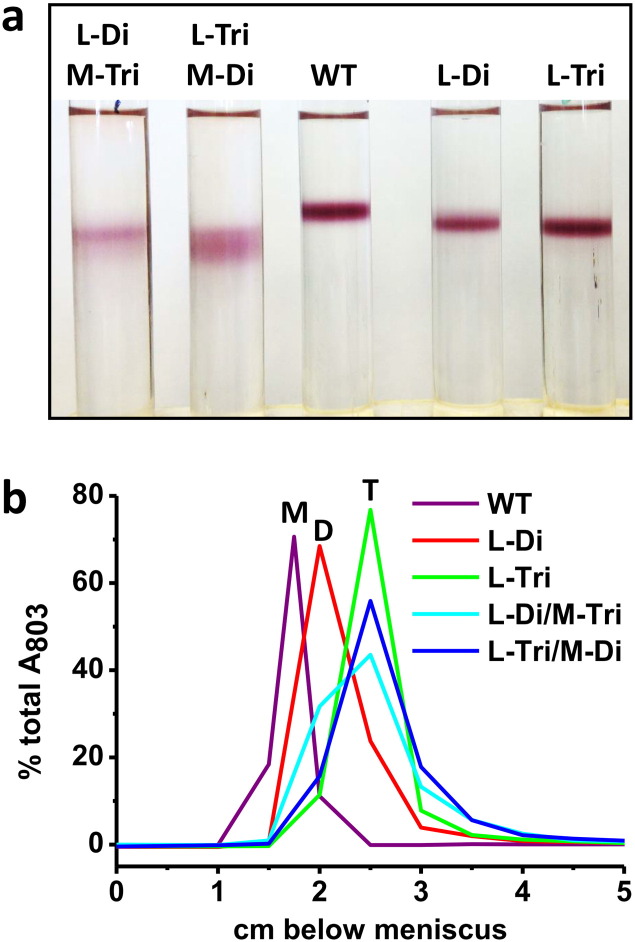
Double PufL/PufM-modified reaction centres assemble as oligomers. (a) Sucrose density gradients of purified proteins. (b) Absorbance at 803 nm in spectra recorded through each gradient at various distances below the meniscus, expressed as a percentage of the total 803 nm absorbance arising from the reaction centre in the gradient. The three main peaks correspond to monomers (M), dimers (D) and trimers (T).

Adding more than one extra-membrane α-helix to an integral membrane protein presents an even greater challenge to the membrane protein assembly machinery. To test whether such a strategy might be feasible, two versions of the *pufL* gene modified with a sequence encoding an α-helix forming a homo-dimeric or homo-trimeric coiled-coil were co-expressed in all possible combinations with two versions of the *pufM* gene modified in the same way. This produced four bacterial strains denoted L-Di/M-Di, L-Di/M-Tri, L-Tri/M-Di and L-Tri/M-Tri; in the case of the L-Di/M-Di RC both the L- and M-polypeptide were modified with the same homo-dimer forming coiled-coil (and so on). Encouragingly, despite the added challenge presented to the assembly machinery, reaction centres exhibiting a normal cofactor absorbance spectrum could be purified from all four strains in the normal way through a combination of nickel-affinity chromatography and gel filtration. Migration of the resulting purified reaction centres on sucrose density gradients consistently demonstrated the presence of complexes that were larger than monomers. In the case of the sample data shown in Fig. 8, both L-Tri-M-Di and L-Di/M-Tri reaction centres migrated as a mixture of dimers and trimers. It was also consistently the case that the pigmented bands formed by these doubly-modified reaction centres were more diffuse than those seen for the WT reaction centre or the L-Di and L-Tri reaction centres used as controls, suggesting a greater structural heterogeneity. Similar data were obtained for the L-Di/M-Di and L-Tri/M-Tri combinations (not shown). The conclusion from this analysis was that double modification with an extra-membrane α-helix did not prevent assembly of the reaction centre despite the challenges presented to the assembly process by tethering component polypeptides together (with the added complexity of the potential to form L/M hetero-dimers in the L-Di/M-Di strain or hetero-trimers in the L-Tri/M-Tri strain). The ability of these and related doubly-modified RCs to form homo-oligomers, hetero-oligomers and arrays, either in membranes or on surfaces, is under ongoing investigation.

## 4 Conclusions

In conclusion, we have shown that the addition of an extra-membrane α-helix with a sequence-dependent propensity to form a coiled-coil bundle can drive the assembly of the purple bacterial reaction centre into a defined, non-native oligomeric form. This approach through fusion of an exposed N- or C-terminus to an extra-membrane component does not necessarily require detailed information on the structure of the target membrane protein, which is of advantage given the relatively slow rate of progress in determining atomic structures for the diversity of integral membrane proteins. An important factor in determining whether this tethering strategy was successful, and the assembly of the monomeric form of the membrane protein was not interfered with, was the length of the linking sequence between the coiled-coil helix and the body of the protein, some trial and error being needed to optimise this. Our conclusion was that the length of this linker not only depended on the spacing needed between the coiled-coil bundle and the attachment point at the N-terminus of the reaction centre polypeptide, but also the space needed to avoid interference with the normal process of monomer assembly.

Encouragingly, oligomer formation proved possible through modification of two of the three component polypeptides of the reaction centre, double modifications were tolerated, and hetero-oligomers could be formed by mixing separately assembled components *in vitro*, suggesting that this strategy of using extra-membrane coiled-coils to driving formation of synthetic oligomers could also be extended to the formation of longer-range arrays. The demonstration that hetero-oligomers can be formed *in vitro* also highlights a route to programmed hetero-oligomerisation of combinations of two or more different membrane proteins, combining mixtures of membrane proteins and soluble proteins, or using a component of a coiled-coil bundle to tether an assembly of one or more (membrane) proteins to a surface or nanomaterial. We are currently exploring the use of this approach for the assembly of hybrid solar energy conversion systems.

Finally, although the study described above focussed on the use of parallel coiled-coils, amino acid sequences are also available for the assembly of antiparallel coiled-coils [39]. If placed appropriately, this opens up the prospect of building programmable three-dimensional arrays of integral membrane proteins. It has been pointed out that a generic strategy for organising proteins into regular geometric structures could have wide-ranging applications in crystallography [74]. Given the specific challenges of membrane protein crystallography, one wonders whether the ability to organise monomeric integral membrane proteins into ordered two- or three-dimensional arrays could have an impact on membrane protein structural biology.

## Transparency document

Transparency document. Image 2

## Figures and Tables

**Table 1:** Mass estimates for PufL-modified oligomeric reaction centres.

Construct	Mass (kDa)			
	Theoretical^a^	Analytical gel filtration^b^	Sedimentation velocity AUC^c^	Equilibrium AUC^d^
WT	104	163	95.8	130 ± 6
L-Di	216	447	219	236 ± 2
L-Tri	324	633	307	375 ± 31
L-Tet	436	734	320	517 ± 30

a Mass estimated from the protein sequence and masses of the cofactors.

b Mass estimated using a calibration curve based on gel filtration standards.

c Mass estimated from sedimentation coefficients in Sedfit.

d Mass determined by curve fitting in Ultrascan II.

## Appendix A Supplementary data

Supplementary material. Image 3
