# Supplementary material for: Directed assembly of defined oligomeric photosynthetic reaction centres through adaptation with programmable extra-membrane coiled-coil interfaces
Source: Biochim Biophys Acta. 2016 Dec;1857(12):1829–39. doi: 10.1016/j.bbabio.2016.09.002 (PMC5084686; doi:10.1016/j.bbabio.2016.09.002)
Supplement: Supplementary file 2 — Supplementary material. [file mmc2.docx]

Supporting Information

**Directed assembly of defined oligomeric photosynthetic reaction centres through adaptation with programmable extra-membrane coiled-coil interfaces**

David J. K. Swainsbury^a,c^, Robert L. Harniman^b^, Natalie D. Di Bartolo^a^, Juntai Liu^a^, William F.M. Harper^a^, Alexander S. Corrie^a^ and Michael R. Jones^a,^*

^a^ *School of Biochemistry, Medical Sciences Building, University of Bristol, University Walk, Bristol BS8 1TD, United Kingdom*

^b^ *School of Chemistry, University of Bristol, Cantock's Close, Bristol BS8 1TS, United Kingdom*

^c^ *Present address: Department of Molecular Biology and Biotechnology, University of Shefﬁeld, Shefﬁeld S10 2TN, United Kingdom*

*Corresponding author

email: m.r.jones@bristol.ac.uk

**Supporting Methods and Results**

*1. Modification of PufL.*

Organisation of the naturally-overlapping reaction centre *pufL* and *pufM* genes is shown in Fig. S2a. Protein sequences encoding a coiled-coil (blue) and linker (yellow) were inserted between the N-terminal methionine and residue 1 of PufL (alanine), by modification of *pufL* (Fig. S2b). The *pufL* and *pufM* genes overlap by 8 base pairs such that the Shine-Dalgarno sequence and start codon for *pufM* are embedded in *pufL* (Fig. S2c, top).

The C-terminus of PufM was modified by the sequence LALVPRGSSAHHHHHHHHHH to introduce a thrombin cleavage site (underlined) and a ten histidine tag to assist purification (Fig. S2a, orange).

*2. Modification of PufM.*

For PufM (Fig. S2d,e), protein sequences encoding a coiled-coil (blue) and a linker (yellow) were also inserted between the N-terminal methionine and residue alanine 1 (Fig. S2d). Modification of the N-terminus of PufM required disentangling the 3' end of *pufL* and the 5' end of *pufM* prior to the introduction of the coiled-coiled sequence. This was achieved as shown in Fig. S2c. Bases in lower case indicate silent changes to the coding sequence for *pufL* to facilitate the separation, including removal of a *Sma*I restriction site and the unwanted native Shine-Dalgarno sequence for *pufM*.

In this construct the C-terminus of PufM was also modified by a thrombin cleavage site and ten histidine tag (Fig. S2e, orange).

*3. Reaction centre purification.*

As described in detail elsewhere [S1], photosynthetic membranes were prepared from **c**ells grown under dark/semiaerobic conditions, and reaction centres isolated from these were purified by nickel affinity chromatography with 0.5 % n-dodecyl-N,N-dimethylamine-N-oxide (LDAO) as the solubilising detergent. Reaction centre were detergent exchanged into n-dodecyl β-D-maltopyranoside (DDM) by gel filtration in 20 mM Tris (pH 8)/200 mM NaCl/0.04 % (w/v) DDM (RC-DDM buffer) on a Superdex 200 16/600 column (GE Healthcare). Reaction centres were used for subsequent experiments as concentrated solutions in RC-DDM buffer unless stated otherwise.

The purity of reaction centres was assessed by UV/Vis absorbance spectroscopy [S2], fractions with a ratio of protein absorbance at 280 nm to bacteriochlorophyll absorbance at 802 nm of less than 1.4 being retained for use. Reaction centre concentrations were calculated using an extinction coefficient of 2.88 × 10^5^ M^-1^ cm^-1^ at 802 nm [S3]. Absorbance spectra were recorded in RC-DDM buffer in the presence of 2 mM **s**odium ascorbate to counteract oxidative bleaching of the reaction centre absorbance band at 865 nm.

*4. Sidedness of reconstituted oligomeric reaction centres.*

Attempts were made to quantify the sidedness of reaction centre reconstitution into liposomes by tryptic digestion to release the His_10_ tag from reaction centres in intact and detergent disrupted liposomes (data not shown). Loss of the His_10_ tag was quantified by a shift to lower molecular weight of a band corresponding to PufM separated from that of PufL and PuhA by SDS-PAGE. This assay yielded results for WT reaction centres consistent with estimates produced previously by others [S4], with ~60 % of reaction centres oriented with the P BChls exposed to the external phase. The same assay was repeated with the L-Di, L-Tri and L-Tet reaction centres but a clear result could not be obtained due to the increase in mass for PufL caused by the coiled coil resulting in overlap between the bands for the modified PufL and the tryptic digest of PufM. Nevertheless there was some indication that the degree of digestion of PufM was greater for these reaction centres, which would correspond to an increase in the percentage of reaction centres with the P BChls exposed to the exterior of the liposome. As the coiled-coil is located on the opposite side of the reaction centre, such a change could indicate an interaction between the coiled-coil domain and the membrane that affects the sidedness of reconstitution. Such an interaction could also help to explain the consistently greater efficiency of reconstitution of L-Tri and L-Tet reaction centres relative to the WT reaction centre. We are currently developing an improved assay to investigate this further.

*5. Molecular dynamics simulations.*

Energy minimized molecular models of oligomers were based on PDB structures for the reaction centre (PDB ID: 2J8C [S5]) and homo-dimer, trimer and tetramer coiled-coils (PDBIDs: 4DZM, 4DZL and 3R4A [S6]). Using PyMOL, for each oligomer the appropriate number of reaction centres were arranged around the coiled-coil structure to bring the N-terminus of the reaction centre PufL polypeptide as close as possible to the C-terminus of one of the α-helices of the coiled-coil without clashes. Linker sequences were added and reshaped using the sculpting tool to avoid clashes with the PuhA polypeptide. The result was a single coiled-coil-linker-PufL chain. To simplify the simulations, cofactors were removed from the reaction centre after demonstrating that this had little effect on the structure of the WT reaction centre during simulation (see below).

The environment for the reaction centres was a 128 molecule 1-palmitoyl-2-oleoyl-*sn*-glycero-3-phosphocholine (POPC) bilayer and corresponding parameter file [S7] that was expanded either two by two for 512 lipids (WT) or three by three for 1156 lipids (multimers) with the GROMACS 5.0 genconf tool [S8]. Reaction centres were manually aligned within the bilayer with Swiss PDB viewer [S9], and structures were then parameterized using a modified version of the GROMOS 53a6 force field [S10] updated to include Berger lipids [S11] and using the single point charge water model. The system was solvated and reaction centre proteins were embedded in the membrane using the g_membed tool [S12] with a scaling factor of 0.5 (WT, dimer and trimer) or 0.65 (tetramer). Random water molecules were replaced with the appropriate number of sodium ions to give a net charge of zero. Each system was energy minimized to less than 1000 kJ/mol/nm over 50,000 steps. They were then equilibrated as an NPT ensemble for 1 ns using gmx mdrun at a temperature of 300 K and semi-isotropic pressure coupling at 1 bar in both the x, y and z dimensions with protein heavy atoms restrained.

Simulations were run as NPT ensembles over 50,000,000 steps of 0.002 ps giving a total simulation time of 100 ns. The reference temperature was 300 K and semi-isotropic pressure coupling was used at 1 bar in both the x + y, and z dimensions. For each system three simulations were performed using a random seed to generate initial velocities. All simulations were performed using GROMACS 5.0 on BlueCrystal Phase 3 GPU nodes (Advanced Computing Research Centre, University of Bristol). This yielded between 4 and 10 ns/day per node depending on the system being simulated.

Proteins were centred within the simulation box and corrected for periodicity with the pbc mol option in gmx trjconv. Parameters from the repeat simulations are shown in Fig. S6. Plots of the system potential were generated using gmx energy, the backbone root mean square deviation (RMSD) was plotted using gmx rms, the radius of gyration was plotted using gmx gyrate, and the surface accessible surface area and solvent excluded volume of the protein was determined with gmx sasa with the –tv option. Final structures were converted to PDB format and overlays of repeated simulations were produced with backbone alignments using PyMOL.

The overall structure of the WT reaction centre was retained over the course of three repeat simulations despite the removal of the cofactors to simplify the computations. Small movements in some transmembrane helices were observed, particularly the A-helices of PufL and PufM, and a small rotation of the soluble domain of PuhA about the X-axis. Nevertheless variation in the RMSD for the whole WT reaction centre structure during each simulation was less than 5 Å (Fig. S6b, left) and the radius of gyration, solvent excluded volume and solvent accessible surface area (Fig. S6c-e, respectively) were all stable indicating there was no collapse of the ligand binding cavities within the protein. This was also evident from inspection of overlaid models of the WT reaction centre (Fig. S7).

Three simulations were carried out for each oligomer producing similar results in terms of the packing and interactions of the reaction centre monomers (see convergences of parameters in Fig. S6). In all cases (with the exception of the WT reaction centre monomer – see Fig. S6c, left) each simulation resulted in the formation of a more compact structure than in the starting model, the monomers being pulled towards one another and showing limited variation in gross position from simulation to simulation (see overlaid final models in Fig. S8). Compaction was quantified by a consistent decrease in the calculated radius of gyration for the whole structure over each simulation (Fig. S6c) and was obvious for each of the oligomers from a comparison of the starting model and a final model (Fig. S9).

The factor limiting the size of each oligomeric structure was steric clashes between adjacent monomers, but no specific molecular interactions were seen that were reproduced between all adjacent monomers in the oligomer. This is further supported by the unaltered solvent accessible surface area (Fig. S6e) demonstrating that no additional protein surface was buried in the stable final structures despite their more compact nature. Differences in overall structure of the reaction centre subunits were similar to those of WT reaction centre suggesting that the tethering by the coiled-coil did not disrupt the protein fold. The main variation within each set of oligomer structures was the final position of the extra-membrane coiled-coil relative to the plane of the membrane (Fig. S10); in some structures the coiled-coil was orthogonal to the membrane but in others it resided at a more acute angle.

An additional point to note is that the conformation of each reaction centre in each final, more compact model of each oligomer was largely determined by its conformation in the starting structure. This is particularly evident by considering the orientation of the right-most monomer in the starting and final structures of the L-Tet reaction centre assembly in Fig. S9 which was rotated somewhat relative to the other three monomers. We concluded that there was limited rotation of each reaction centre monomer around the axis perpendicular to the plane of the membrane during each simulation, the main movement over the 100 ns simulation rather being a pulling-in of each reaction centre towards the central coiled-coil until steric interactions between monomers prevented further movement. Thus the details of each final model should be treated with caution as its details were strongly biased by the starting model. Future MD simulations will examine the influence of different starting conformations for the reaction centres relative to the coiled-coil. However, in the context of the present study this was not an issue as the function of the modelling was simply to establish whether the overall dimensions and geometry of each energy minimized oligomer was consistent with the objects seen in AFM imaging.

**Supporting References**

[S1] D.J.K. Swainsbury, V.M. Friebe, R.N. Frese, M.R. Jones, Evaluation of a biohybrid photoelectrochemical cell employing the purple bacterial reaction centre as a biosensor for herbicides, Biosens. Bioelectron. 58 (2014) 172–178.

[S2] M.Y. Okamura, M.A. Steiner, G. Feher, Characterization of reaction centers from photosynthetic bacteria .1. Subunit structure of protein mediating primary photochemistry in *Rhodopseudomonas-spheroides* R-26, Biochemistry 13 (1974) 1394–1402.

[S3] S.C. Straley, W.W. Parson, D.C. Mauzerall, R.K. Clayton, Pigment content and molar extinction coefficients of photochemical reaction centers from *Rhodopseudomonas-spheroides*. Biochim. Biophys. Acta 305 (1973) 597–609.

[S4] M. Hara, T. Ueno, T. Fujii, Q. Yang, Y. Asada, J. Miyake, Orientation of photosynthetic reaction center reconstituted in neutral and charged liposomes, Biosci, Biotech. Biochem. 61 (1997) 1577–1579.

[S5] J. Koepke, E.-M. Krammer, A.R. Klingen, P. Sebban, G.M. Ullmann, G. Fritzsch, pH modulates the quinone position in the photosynthetic reaction center from *Rhodobacter sphaeroides* in the neutral and charge separated states, J. Mol. Biol. 371 (2007) 396–409.

[S6] J.M. Fletcher, A.L. Boyle, M. Bruning, G.J. Bartlett, T.L. Vincent, N.R. Zaccai, C.T. Armstrong, E.H.C. Bromley, P.J. Booth, R.L. Brady, A.R. Thomson, D.N. Woolfson, A basis set of *de novo* coiled-coil peptide oligomers for rational protein design and synthetic biology, ACS Synth. Biol. 1 (2012) 240−250.

[S7] D.P. Tieleman, L.R. Forrest, M.S.P. Sansom, H.J.C. Berendsen, Lipid properties and the orientation of aromatic residues in OmpF, influenza M2, and alamethicin systems: Molecular dynamics simulations, Biochemistry 37 (1998) 17554–17561.

[S8] S. Pronk, S. Páll, R. Schulz, P. Larsson, P. Bjelkmar, R. Apostolov, M.R. Shirts, J.C. Smith, P.M. Kasson, D. van der Spoel, B. Hess, E. Lindahl, GROMACS 4.5: a high-throughput and highly parallel open source molecular simulation toolkit, Bioinformatics 29 (2013) 845–854.

[S9] N. Guex, M.C. Peitsch, SWISS-MODEL and the Swiss-PdbViewer: An environment for comparative protein modelling, Electrophoresis 18 (1997) 2714–2723.

[S10] C. Oostenbrink, A. Villa, A.E. Mark, W.F. van Gunsteren, A biomolecular force field based on the free enthalpy of hydration and solvation: The GROMOS force-field parameter sets 53A5 and 53A6. J. Comput. Chem. 25 (2004) 1656–1676.

[S11] O. Berger, O. Edholm, F. Jähnig, Molecular dynamics simulations of a fluid bilayer of dipalmitoylphosphatidylcholine at full hydration, constant pressure, and constant temperature, Biophys. J. 72 (1997) 2002–2013.

[S12] M.G. Wolf, M. Hoefling, C. Aponte-Santamaría, H. Grubmüller, G. Groenhof, g_membed: efficient insertion of a membrane protein into an equilibrated lipid bilayer with minimal perturbation, J. Comput. Chem. 31 (2010) 2169–2174.


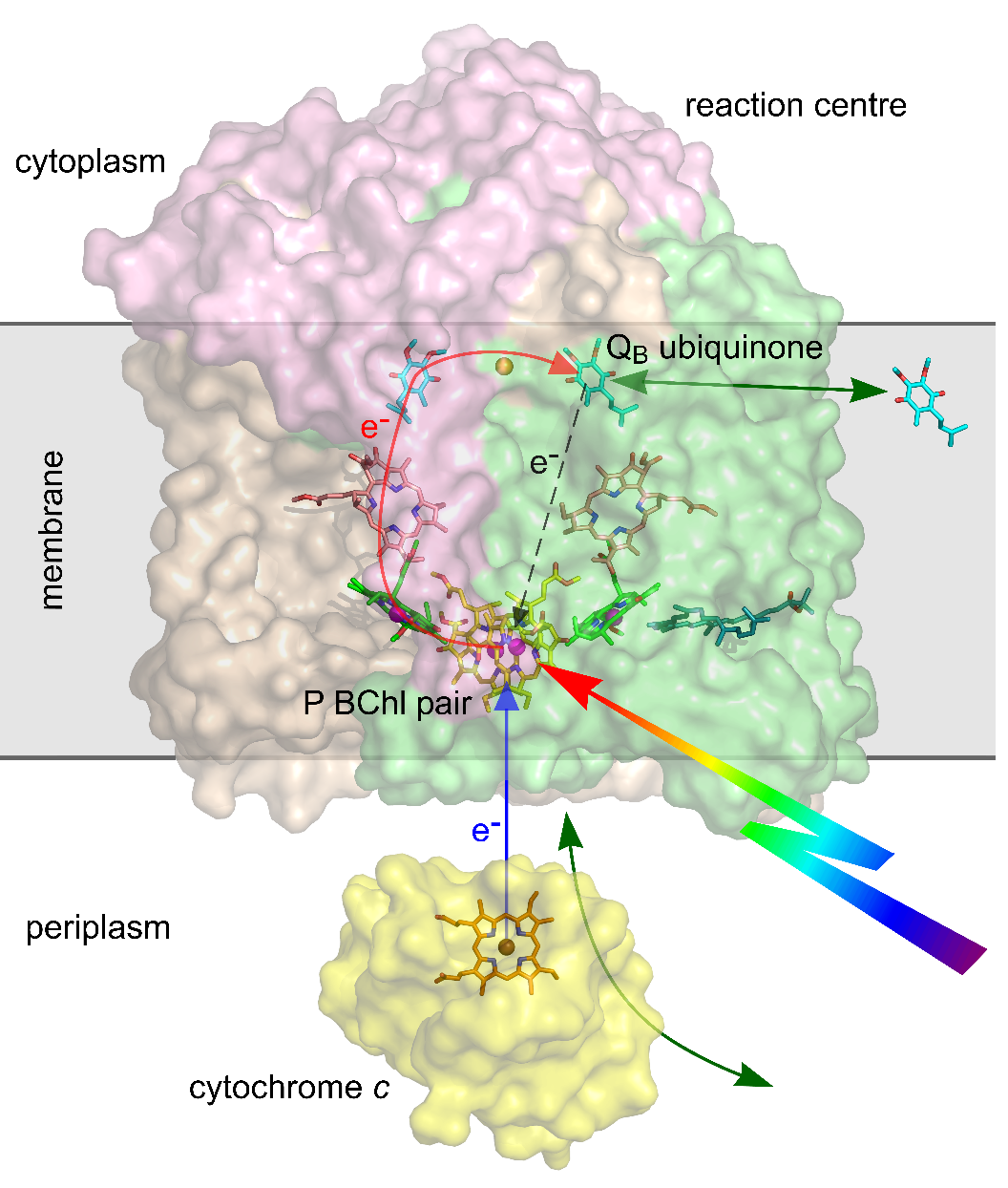


**Fig. S1.** Cofactor structure and mechanism of the *Rba. sphaeroides* reaction centre. Structure: The reaction centre and cyt *c* proteins are shown with semi-transparent surfaces, with the cofactors as sticks. Reaction centre cofactors are the BChl pair (yellow carbons), monomeric BChls (green carbons), bacteriopheophytins (pink carbons), ubiquinones (cyan carbons), carotenoid (turquoise carbons) and Fe atom (brown sphere). Cyt *c* cofactor is haem C (orange carbons). Other atoms are Fe (brown sphere), Mg (magenta sphere), N (blue) and O (red). For clarity the hydrocarbon side chains of the bacteriochlorin and ubiquinone cofactors are not shown. Grey slab shows the approximate position of the membrane.

Mechanism: Photoexcitation initiates ultrafast four step electron transfer from the BChl pair (P) to the Q_B_ ubiquinone (red arrow). In the absence of any additional donor or acceptors the P^+^Q_B_^‑^ radical pair recombines in a few seconds (grey dashed arrow). In the presence of reduced cyt *c* photo-oxidation of P initiates electron transfer (blue arrow) from a cyt *c* docked to the periplasmic face of the reaction centre. Cyt *c* and the Q_B_ ubiquinone (after double reduction/protonation) are mobile components (green double arrows).


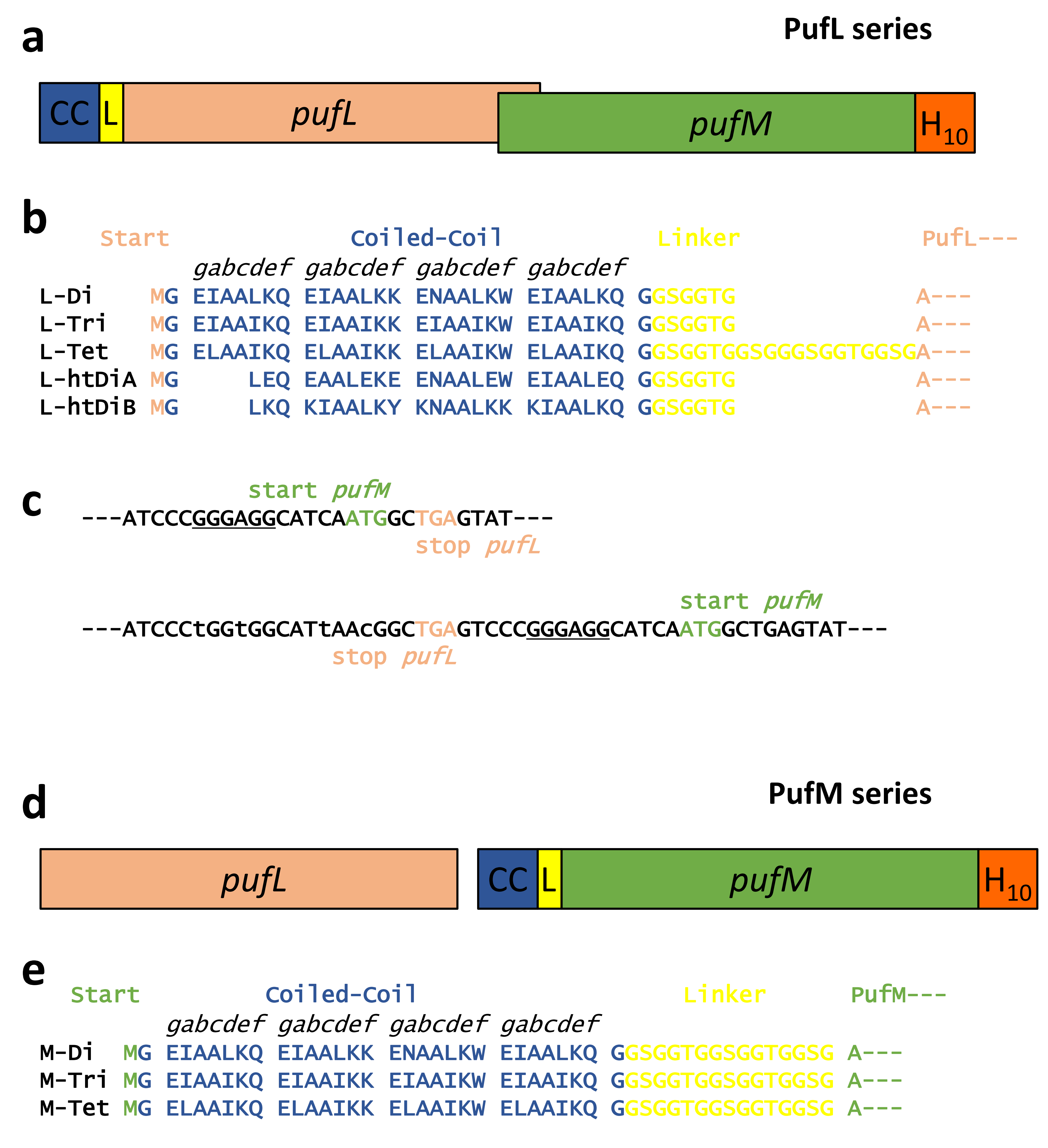


**Fig. S2.** Gene organisation and polypeptide composition. **(a)** For the PufL modified reaction centres the *pufL* gene (beige) was augmented with sequences encoding a coiled-coil polypeptide (blue) and linker (yellow). The overlapping *pufM* gene (green) is modified with a sequence encoding ten histidine residues (orange). **(b)** Coiled-coil sequences (blue) and linkers (yellow) introduced at the N-terminus of PufL (beige). **(c)** Native overlap region between *pufL* and *pufM* (top), and separation of the two genes to facilitate modification of the N-terminus of PufM (bottom). **(d)** For the PufM modified reaction centres the *pufM* gene (green) was modified with sequences encoding a coiled-coil polypeptide (blue), linker (yellow) and ten histidine residues (orange). **(e)** Coiled-coil sequences (blue) and linkers (yellow) introduced at the N-terminus of PufM (green).


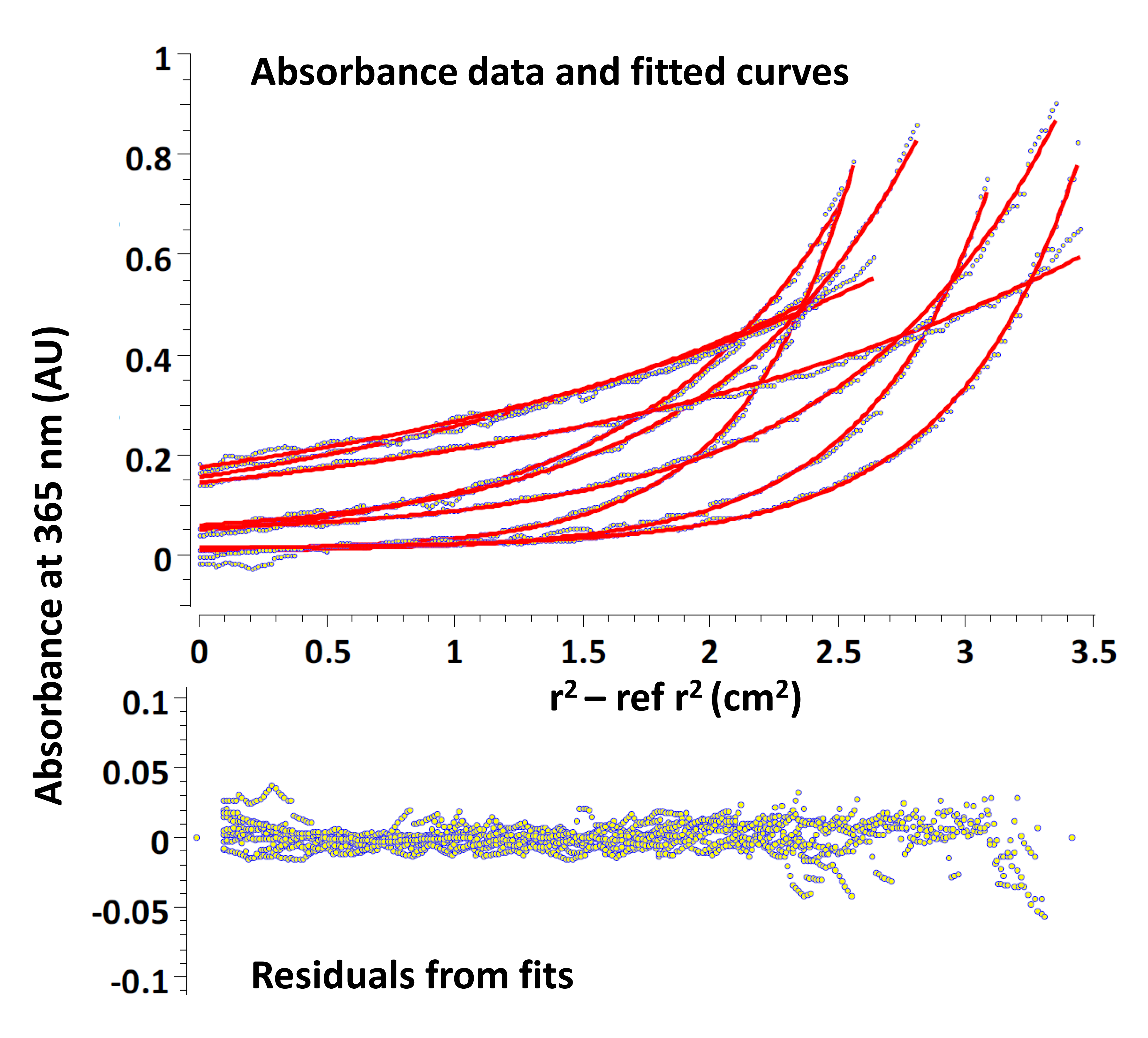


**Fig. S3.** Sample data from equilibrium analytical centrifugation. Absorbance data and fitted curves (top) and residuals (bottom) for L-Tri reaction centres.


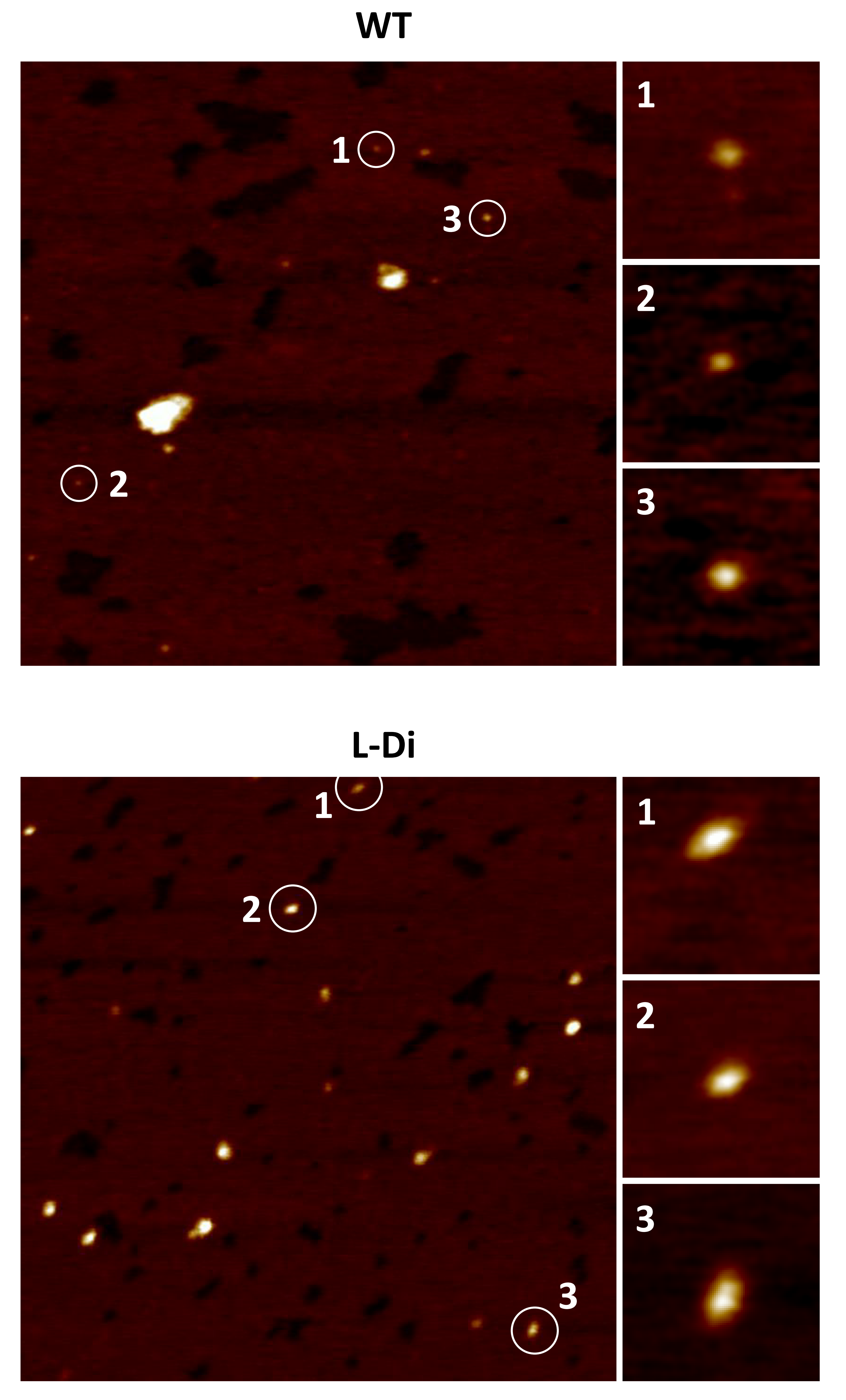


**Fig. S4.** Atomic force microscopy of WT and PufL-modified dimeric reaction centres. Left - widefield view, image dimensions 500 x 500 nm. Right – zoomed view of circled individual complexes, image dimensions 50 x 50 nm.


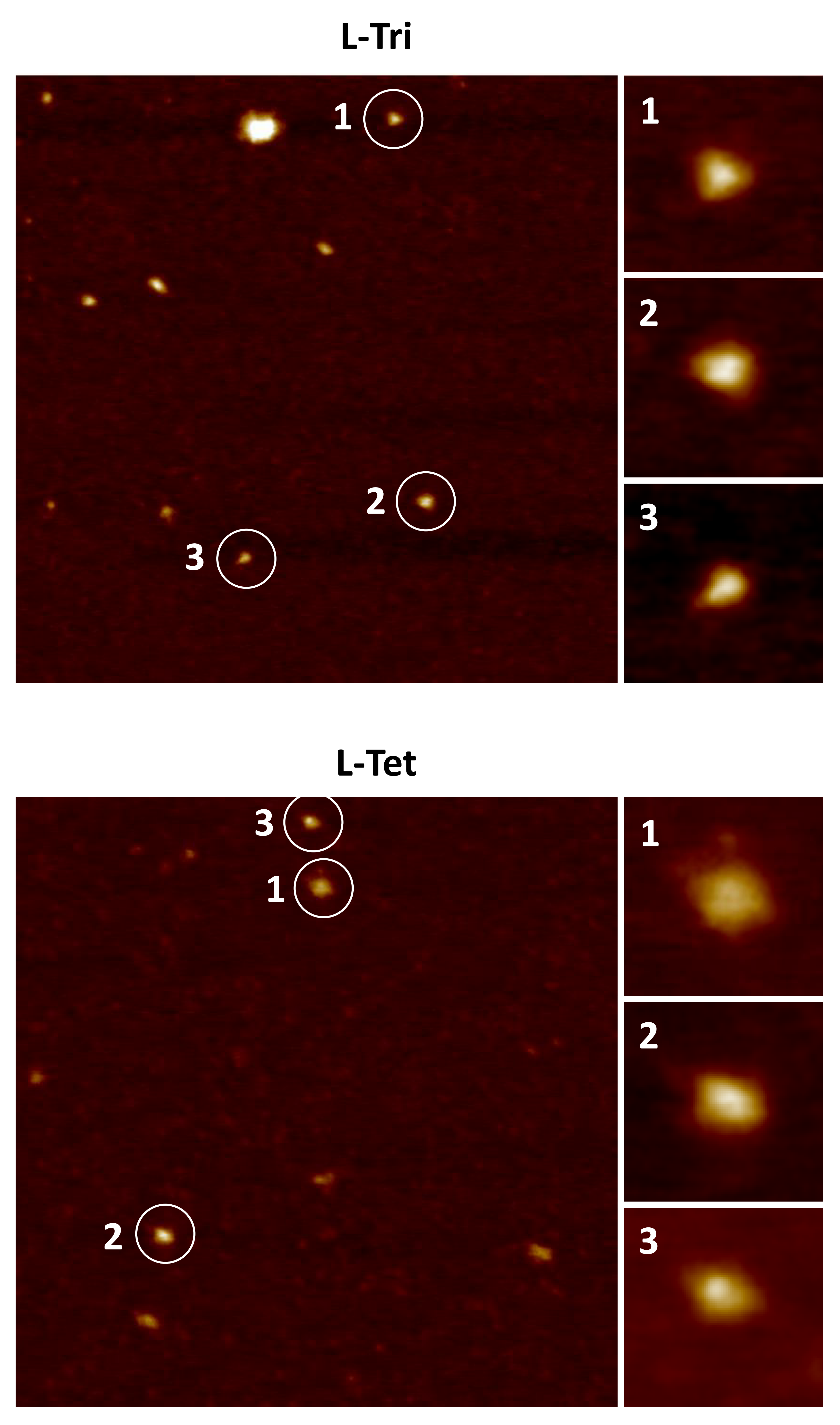


**Figure S5.** Atomic force microscopy of PufL-modified trimeric and tetrameric reaction centres. Left - widefield view, image dimensions 500 x 500 nm. Right – zoomed view of circled individual complexes, image dimensions 50 x 50 nm.


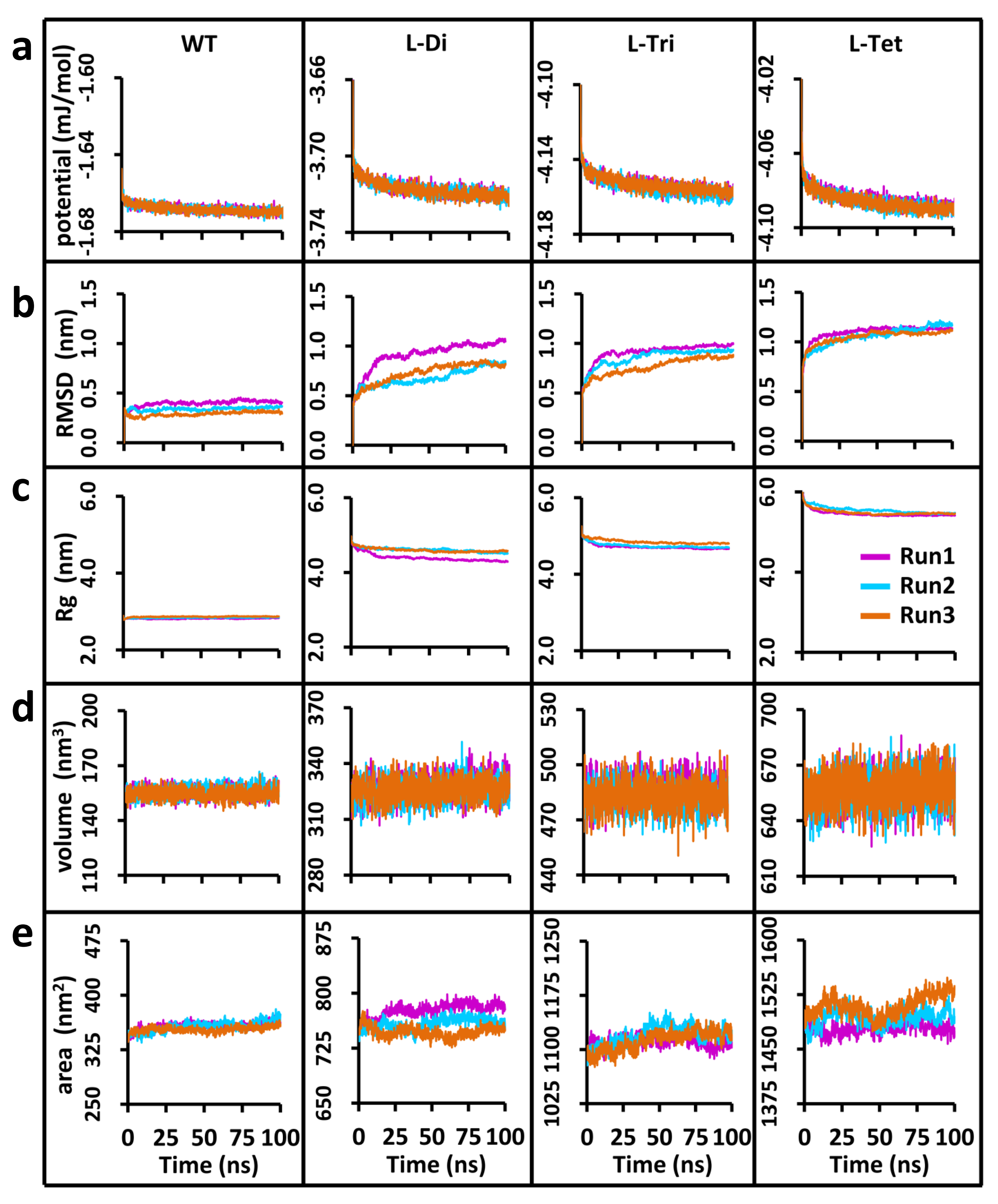


**Fig. S6.** Parameters from molecular dynamics simulations. Plots from three molecular dynamics simulations for each reaction centre: **(a)** system potential; **(b)** protein backbone root mean square deviation (RMSD); **(c)** protein radius of gyration (R_g_); **(d)** solvent excluded volume; **(e)** solvent accessible surface area.


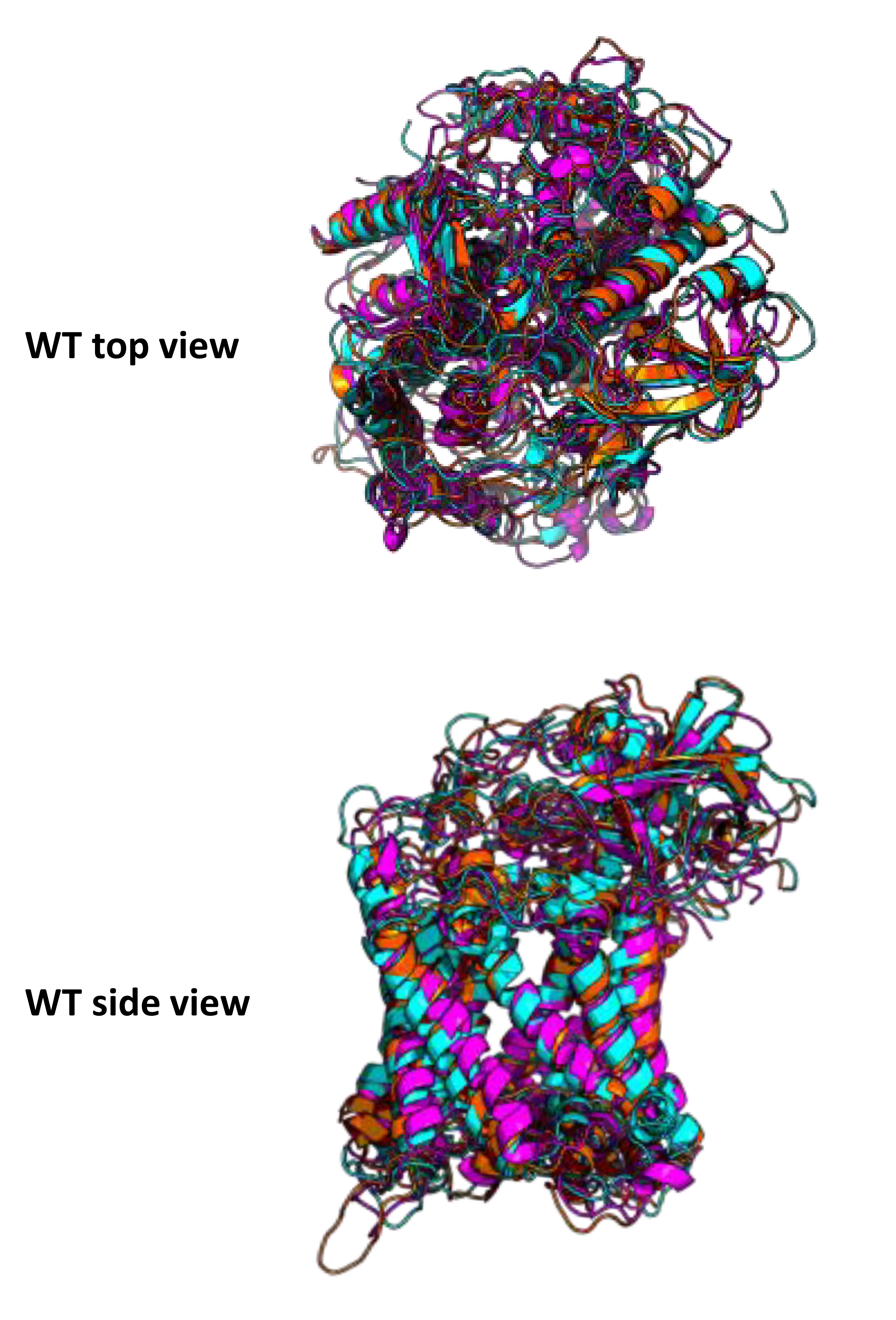


**Fig. S7.** Overlay of three energy-minimized structures for the WT reaction centre. **(a)** top view (orthogonal to the cytoplasmic side of the membrane) and **(b)** side-view (in the plane of the membrane) of ribbon structures of the three final energy-minimized models for the WT reaction centre, colored orange, cyan and magenta.


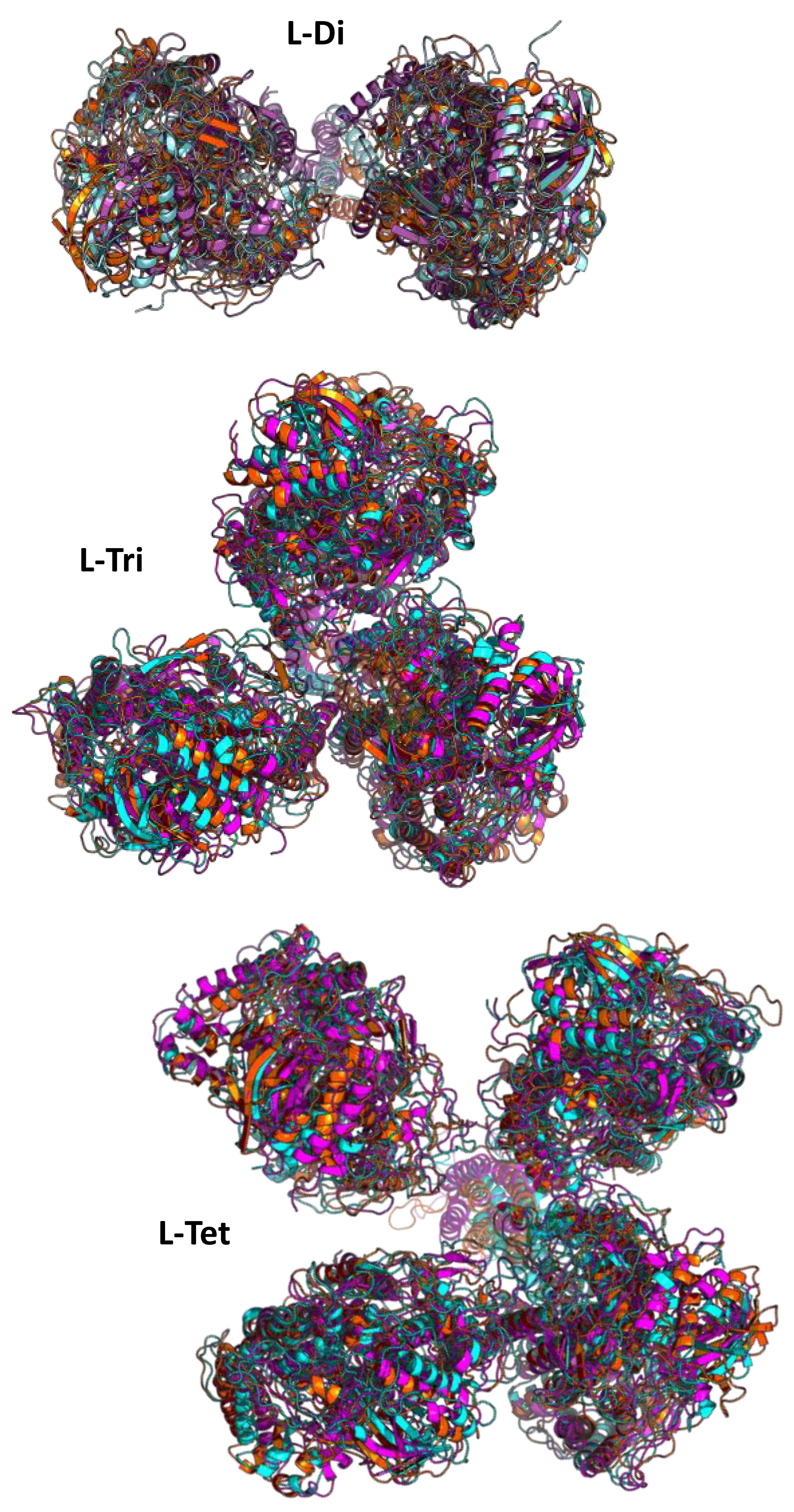


**Fig. S8.** Overlay of the molecular dynamics simulations of the PufL-modified oligomeric reaction centres. For each complex an overlay of the three final energy-minimized models are shown, colored orange, cyan and magenta. The central coiled-coil has been made semi-transparent, and the view is orthogonal to the cytoplasmic side of the membrane.


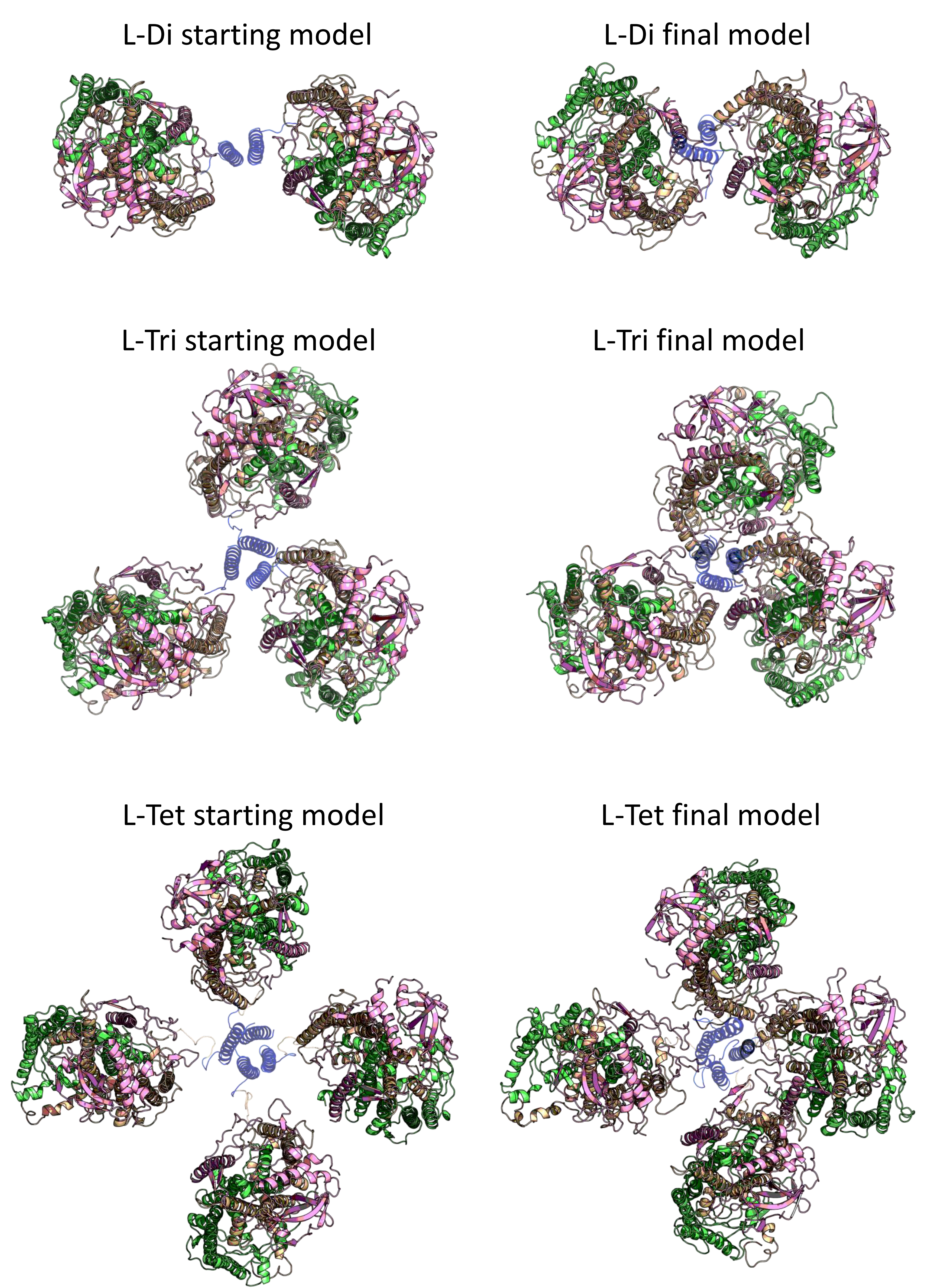


**Fig. S9.** Comparison of starting and final models for PufL-modified oligomeric reaction centres. For each complex a cytoplasmic view of the starting model used for molecular dynamics simulations is compared to the same view of a more compact final model after energy minimisation and 100 ns of simulation. The coiled-coil is shown in blue with the reaction centre polypeptides in beige (PufL), green (PufM) and pink (PuhA).


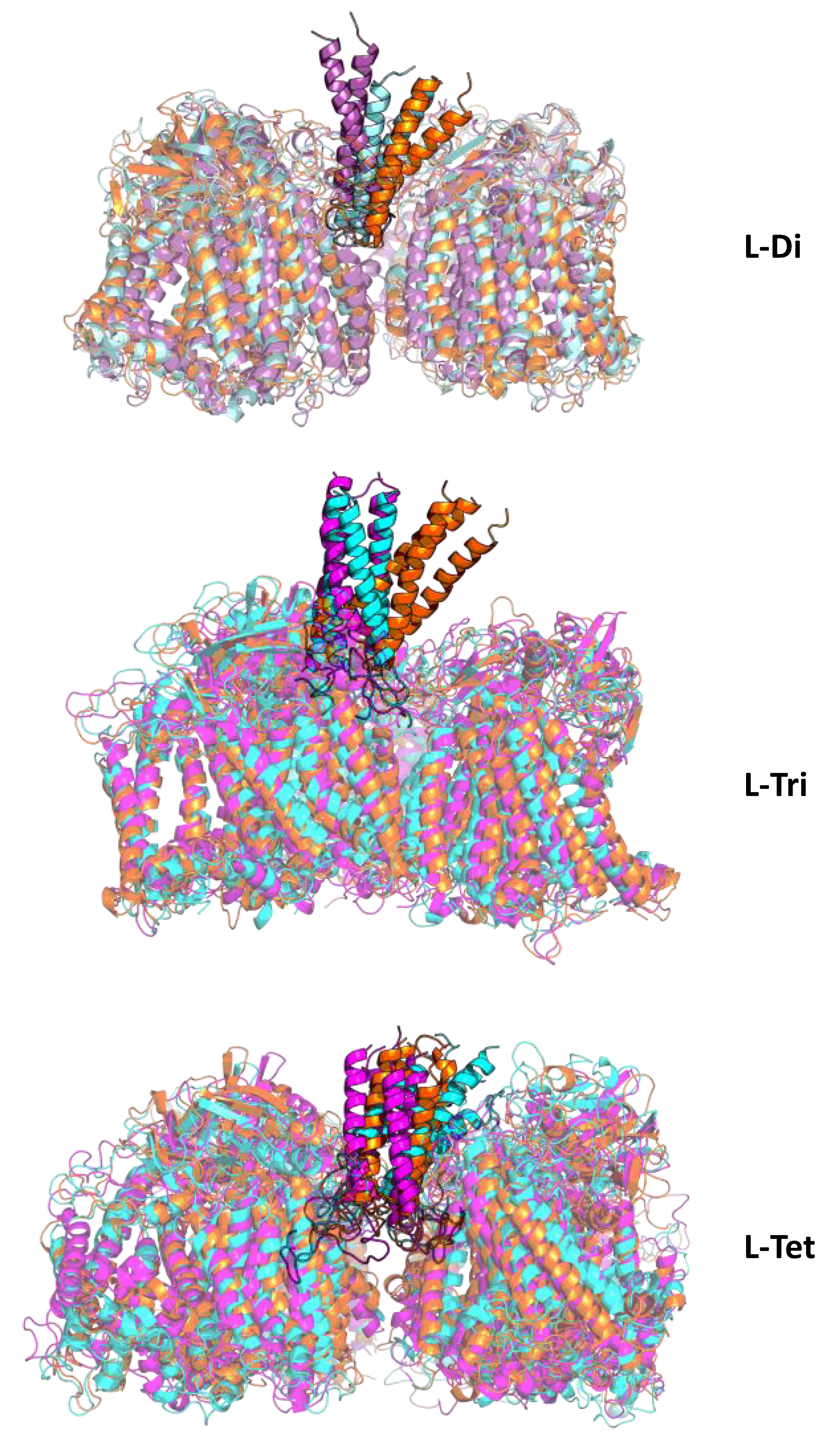


**Fig. S10.** Comparison of the orientation of the coiled-coil module relative to the membrane-embedded reaction centres. For each complex an overlay of the three final energy-minimized models are shown, colored orange, cyan and magenta. The reaction centres have been made semi-transparent to highlight the coiled-coil module. View is approximately in the plane of the membrane.


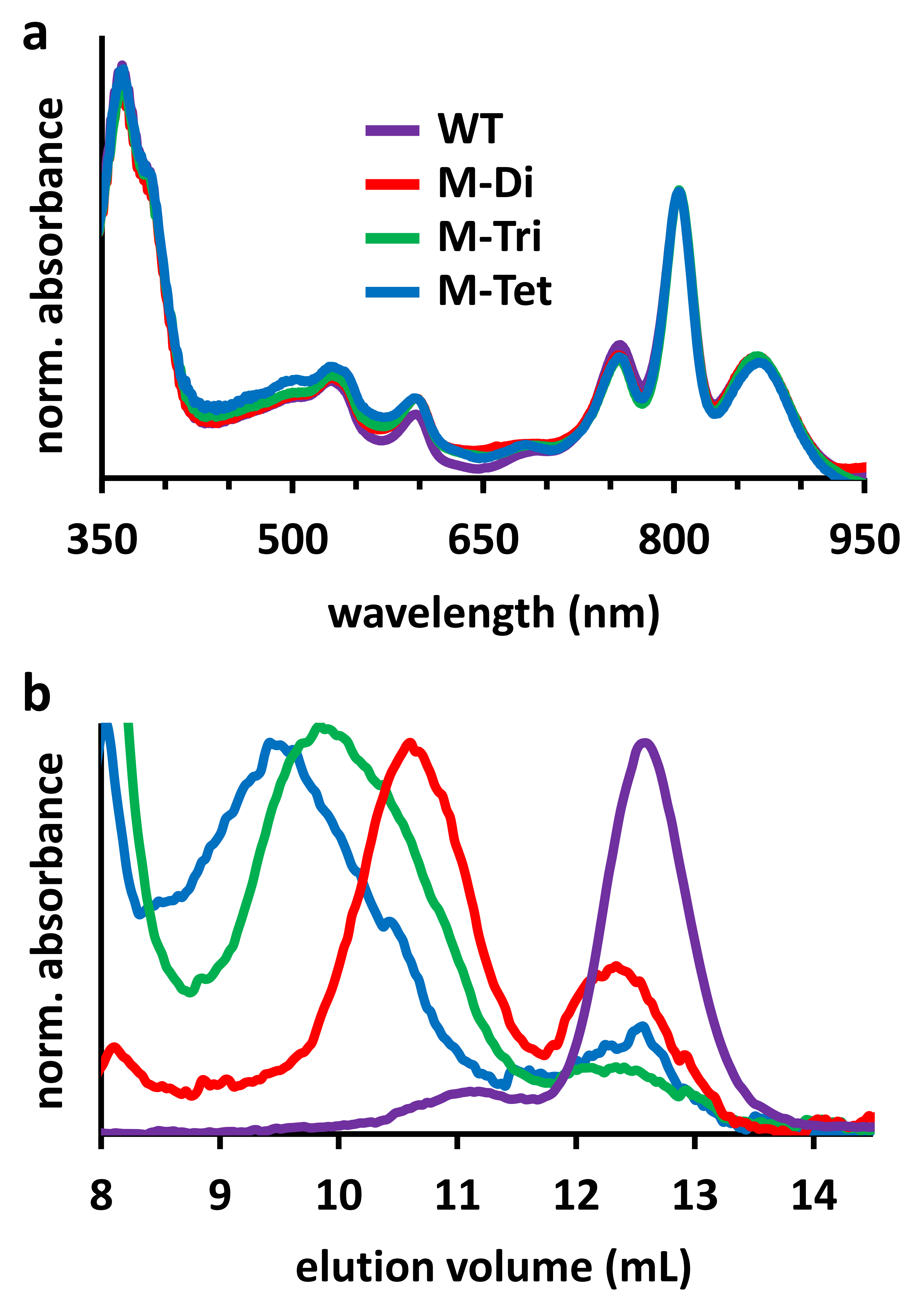


**Fig. S11.** Structural integrity and oligomeric state of PufM-modified reaction centres. **(a)** Absorbance spectra of purified PufM-modified reaction centres, normalized to the same absorbance at 800 nm. **(b)** Analytical gel filtration of purified PufM-modified reaction centres. Masses were calculated from a calibration curve of known standards, and were 164 kDa (WT), 433 kDa (M-Di), 676 kDa (M-Tri) and 785 kDa (M-Tet).


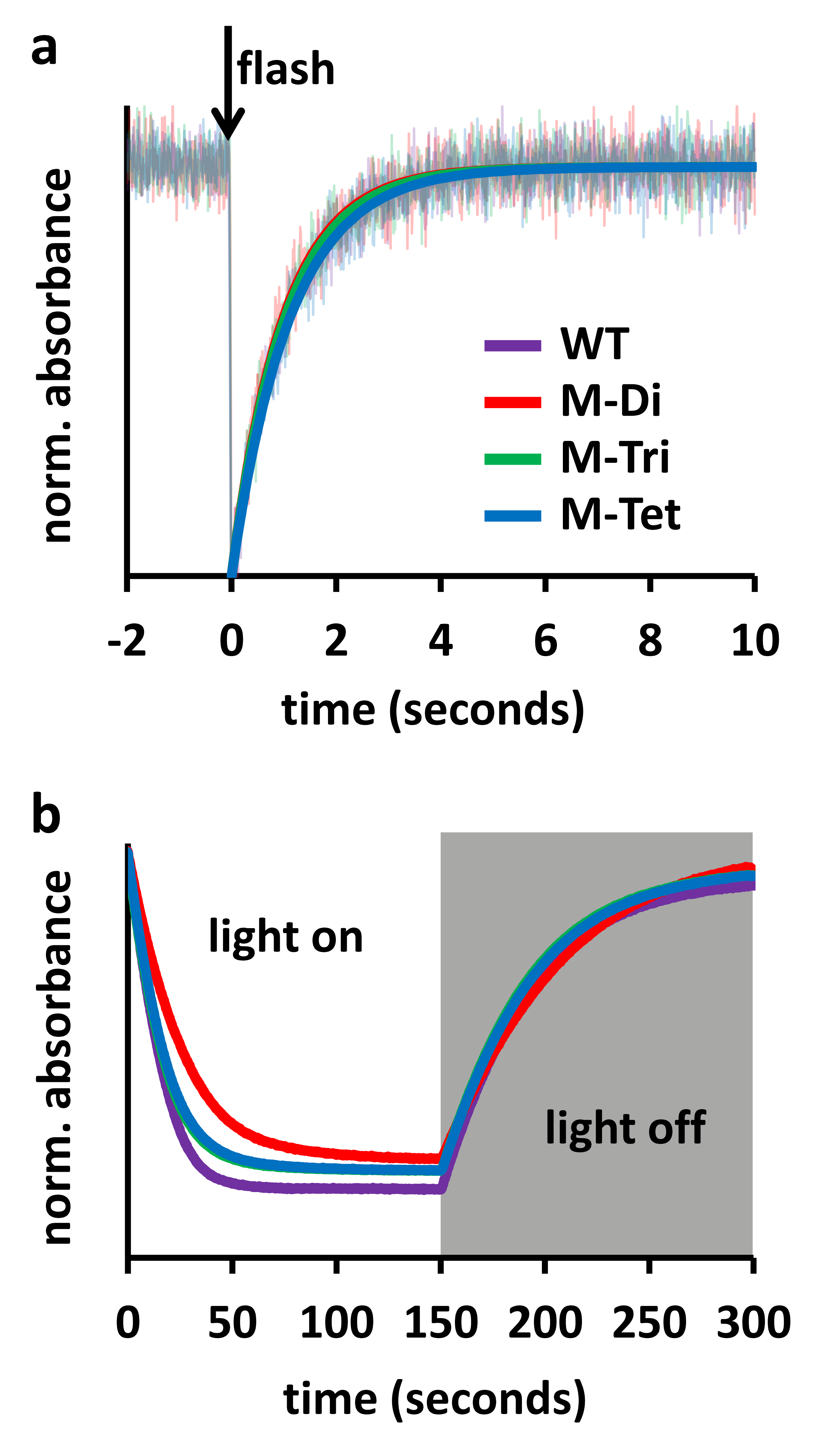


**Fig. S12.** Functional integrity of PufM-modified reaction centres. **(a)** Kinetics of P^+^Q_B_^-^ charge recombination monitored at 865 nm. Normalized averages from eight individual kinetic traces are shown in faded lines overlaid with fits to a single exponential decay function. Position of the excitation flash is indicated by a downward arrow. **(b)** Oxidation of cyt *c* by reaction centres during 150 s of continuous illumination monitored at 550 nm. The dark decay is due to re-reduction of cyt *c*. All traces the average of four individual measurements.


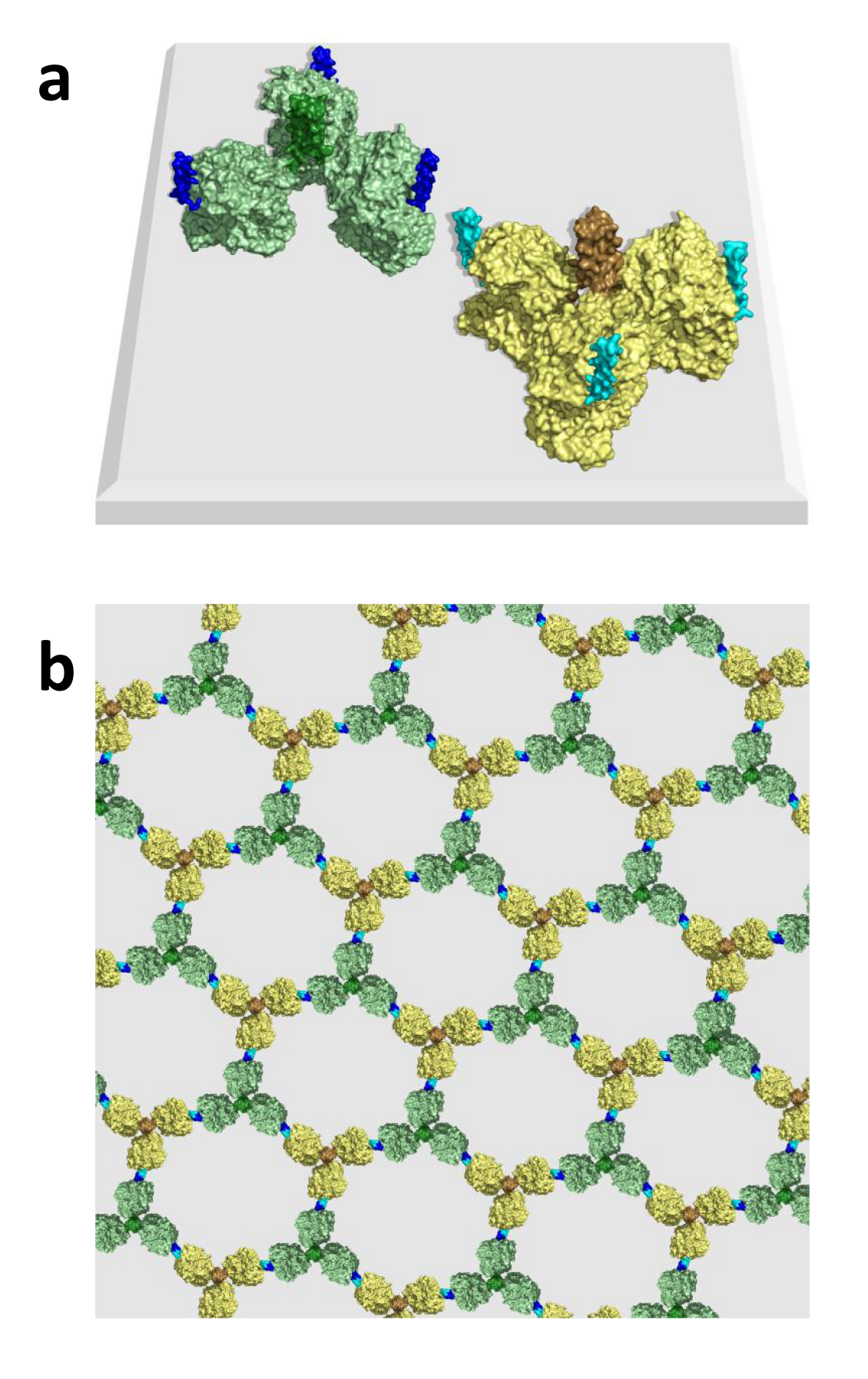


**Fig. S13.** Schematic of array formation by reaction centres modified with two orthogonal coiled-coils. **(a)** Component parts comprised a PufL-modified homotrimer (green reaction centres and dark-green coiled-coil) modified on PufM with half of a hetero-dimeric coiled-coil (blue) and a second homotrimer (yellow reaction centres and brown coiled-coil) modified on PufM with the complementary hetero-dimeric coiled-coil (cyan). **(b)** Schematic of the assembly of an array of the two homotrimers on a surface, exploiting the two-fold symmetry of PufL/PufM.
